# Understanding the Variability in Graph Data Sets through Statistical Modeling on the Stiefel Manifold

**DOI:** 10.3390/e23040490

**Published:** 2021-04-20

**Authors:** Clément Mantoux, Baptiste Couvy-Duchesne, Federica Cacciamani, Stéphane Epelbaum, Stanley Durrleman, Stéphanie Allassonnière

**Affiliations:** 1 ARAMIS Project Team, Inria, 75013 Paris, France; baptiste.couvy@icm-institute.org (B.-C.D.); federica.cacciamani@icm-institute.org (F.C.); stephane.epelbaum@icm-institute.org (S.E.); stanley.durrleman@inria.fr (S.D.); 2 ARAMIS Lab, Brain and Spine Institute, ICM, INSERM UMR 1127, CNRS UMR 7225, Sorbonne University, Hôpital de la Pitié-Salpêtrière, 75013 Paris, France; 3 CMAP, École Polytechnique, 91120 Palaiseau, France; 4 Institute of Memory and Alzheimer’s Disease (IM2A), Centre of Excellence of Neurodegenerative Disease (CoEN), CIC Neurosciences, AP-HP, Department of Neurology, Hôpital de la Pitié-Salpêtrière, 75013 Paris, France; 5 Centre de Recherche des Cordeliers, Université de Paris, INSERM UMR 1138, Sorbonne Université, 75006 Paris, France; stephanie.allassonniere@parisdescartes.fr; 6 HEKA Project Team, Inria, 75006 Paris, France

**Keywords:** network modeling, network variability, Stiefel manifold, MCMC-SAEM, data imputation

## Abstract

Network analysis provides a rich framework to model complex phenomena, such as human brain connectivity. It has proven efficient to understand their natural properties and design predictive models. In this paper, we study the variability within groups of networks, i.e., the structure of connection similarities and differences across a set of networks. We propose a statistical framework to model these variations based on manifold-valued latent factors. Each network adjacency matrix is decomposed as a weighted sum of matrix patterns with rank one. Each pattern is described as a random perturbation of a dictionary element. As a hierarchical statistical model, it enables the analysis of heterogeneous populations of adjacency matrices using mixtures. Our framework can also be used to infer the weight of missing edges. We estimate the parameters of the model using an Expectation-Maximization-based algorithm. Experimenting on synthetic data, we show that the algorithm is able to accurately estimate the latent structure in both low and high dimensions. We apply our model on a large data set of functional brain connectivity matrices from the UK Biobank. Our results suggest that the proposed model accurately describes the complex variability in the data set with a small number of degrees of freedom.

## 1. Introduction

Network science is at the core of an ever-growing range of applications. Network analysis [1] aims at studying the natural properties of complex systems of interacting components or individuals through their connections. It provides a large number of tools to detect communities [2], predict unknown connections [3] and covariates [4], measure population characteristics [5,6] or build unsupervised low-dimensional representations [7]. The need to understand and model networks arises in multiple fields, such as social networks analysis [8], recommender systems [9], gene interactions networks [10], neuroscience [11] or chemistry [12]. Network analysis allows accounting for very diverse phenomenons in similar mathematical frameworks, which lend themselves to theoretical and statistical analysis [13]. In this paper, we are interested in groups of undirected networks that are defined on the same set of nodes. This situation describes the longitudinal evolution of a given network throughout time or the case where the nodes define a standard structure identical across the networks. The former is of interest in computational social science, which studies the evolution of interactions within a fixed population [14]. The latter arises naturally in neuroscience, where the connections between well-defined brain regions are studied on large groups of subjects. The analysis of brain networks is the main application of the present study. It has proven an efficient tool to discover new aspects of the anatomy and function of the human brain [15] and remains a very active research topic [16].

In this study, we are interested in the variability of undirected graph data sets, i.e., how graphs defined on a common set of nodes vary from one network to another. Accounting for this variability is a crucial issue in neuroscience: predicting neurodegenerative diseases or understanding the complex mechanisms of aging requires robust, coherent statistical frameworks that model the diversity among a population. Working on such graphs sharing the same nodes allows comparing them to one another through their adjacency matrices.

The comparison and statistical modeling of such matrices are difficult problems. If all the graphs have *n* nodes, a Gaussian model on the n×n adjacency matrices has a covariance matrix with $n^{4}$ coefficients, which is hard to interpret and difficult to estimate from a reasonable number of observations. Considering adjacency matrices as large vectors allows using classical statistical methods, such as Principal Component Analysis (PCA), but does not take advantage of the strong structures underlying the interactions between the nodes. Tailored kernel methods can be employed to evaluate distances between networks, but many theoretically interesting graph kernels require solving NP-hard problems [17]. In the field of brain network analysis, graphs are often modeled and summarized by features like the average shortest path length, which only partially characterize their structure [6]. Recent methods relying on graphs neural networks often consider the nodes of the network to be permutation invariant, whereas nodes in brain networks play a specific role likely to remain stable across subjects [15,18].

In this paper, we propose a generative statistical model to express the variability in undirected graph data sets. We decompose the network adjacency matrices as a weighted sum of orthonormal matrix patterns with rank one. The patterns and their weights vary around their mean values. Using rank-one patterns allows understanding each decomposition term, while using only a small number of parameters. This is comparable to PCA where each observation is decomposed onto orthogonal elements, which in this case would be matrices. The orthogonal patterns are seen as elements of the Stiefel manifold of rectangular matrices *X* such that $X^{⊤}X$ is the identity matrix [19]. This model allows us to use known distributions and perform a statistical estimation of the mean patterns and weights. We use a restricted number of patterns to get a robust model, which captures the main structures and their variations. This low-dimensional parametric representation provides a simple interpretation of the structure and the variability of the distribution. Our model accounts for two sources of variability: the perturbations of the patterns and their weight. In contrast, current approaches in the literature only consider one of them, as with dictionary-based models and graph auto-encoders.

The proposed framework is expressed as a generative statistical model so that it can easily be generalized to analyze heterogeneous populations. This corresponds to a mixture of several copies of the former model where each cluster has its own center and variance parameters.

In Section 2, we recall relevant literature references for network modeling and statistics on the Stiefel manifold. Section 3 defines our model and further motivates its structure. Section 4 proposes an algorithm based on Expectation-Maximization (EM) to perform Maximum Likelihood Estimation of the model parameters. In Section 5, we present numerical experiments on synthetic and real data. We use our model to predict missing links using the parameters given by the algorithm. We show how our model can be used to perform clustering on network data sets, allowing to distinguish different modes of variability better than a classical clustering algorithm. Applying our method to the UK Biobank collection of brain functional connectivity networks, we demonstrate that our model is able to capture a complex variability with a limited number of parameters. Note that the tools we present here could also be used on any type of network, such as the ones we mentioned above or gene interaction networks.

## 2. Background

### 2.1. Statistical Modeling for Graphs Data Sets

The analysis of graph data sets is a wide area of research that overlaps with many application domains. In this section, we review the principal trends of this field that are used in statistics and machine learning.

The first category of statistical models characterizes graphs in a data set (with possibly varying number of nodes) by a set of features that can be compared across networks, rather than matching the nodes of one graph to those of another. These features can be, for example, the average shortest path length, the clustering coefficient, or the occurrence number of certain patterns. Two examples of such models are Exponential Random Graphs Models [20] and graph kernel methods [17]. Other models are defined by a simple, interpretable generative procedure that allows testing hypotheses on complex networks. The Erdős–Rényi model [21] assumes that each node has an equal probability of connecting with one another. The Stochastic Block Model (SBM, [22]) extends this model and introduces communities organized in distinct clusters with simple interactions. In the limit of a large number of nodes, the same idea gives rise to the graphon model, which has also recently been used to model graph data sets [23]. Finally, recent machine learning models leverage the power of graph neural networks [24] to perform classification or regression tasks. They are used, for instance, in brain network analysis to predict whether a patient is affected by Alzheimer’s disease or how the disease will evolve [25,26].

In this paper, we consider undirected graphs on a fixed given set of *n* nodes connected by weighted or binary edges. This situation arises when studying the evolution of a given network across time [27] or when considering several subjects whose networks have the same structure, for instance, brain networks and protein or gene interaction networks. This constraint allows building models based on the ideas of mean and covariance of adjacency matrices, otherwise ill-defined when the nodes change across networks. In particular, little work has been done in the literature so far on the analysis of the variability of graphs in a data set sharing a common set of nodes. Dictionary-based graph analysis models [28] and graph auto-encoders [25,29] are interesting frameworks in that regard. They allow concisely representing a network in a form that compresses the $O(n^{2})$ adjacency matrix representation into a smaller space of dimension O(p) or O(np) (where *p* is the encoding dimension that characterizes the model). However, they each focus on one aspect of the variability of graph data sets, either the variations of patterns for graph auto-encoders or the variations of patterns weights for dictionary-based models. The model proposed in Section 3 builds on these ideas and accounts for both sources of variability in two latent variables that are combined to obtain the adjacency matrices. These variables are the dominant eigenvalues and the related eigenvectors.

These eigenvectors are regrouped in matrices with orthonormal columns, which makes them points on the Stiefel manifold introduced in the next section. Statistical modeling of these matrices requires taking their geometry into account with manifold-valued distributions.

### 2.2. Models and Algorithms on the Stiefel Manifold

#### 2.2.1. Compact Stiefel Manifolds of Orthonormal Frames

In this paper, we will be considering latent variables belonging to the compact Stiefel manifold $V_{n,p}$, defined as the set of *n*-dimensional orthonormal *p*-frames (with p≤n): $V_{n,p}=\left\{X\in R^{n\times p}|X^{⊤}X=I_{p}\right\}$. Since an element of $V_{n,p}$ can be obtained by taking the *p* first columns of an orthogonal matrix, the Stiefel manifold can be seen as a quotient manifold from the orthogonal group, and thus inherits a canonical Riemannian manifold structure. A detailed and clear introduction to algorithms for optimization and geodesic path computation on the Stiefel Manifold can be found in [30]. More recently, Zimmermann [31] proposed an algorithm to compute the Riemannian logarithm associated with the canonical metric, solving the inverse problem of the geodesic computation.

#### 2.2.2. Von Mises–Fisher Distributions

Various difficulties arise when dealing with statistical distributions on Riemannian manifolds: for instance, computing the barycenter of a set of points can be a difficult problem, if not even ill-posed. The normalizing constant of a distribution is often impossible to compute analytically from its non-normalized density, so Maximum Likelihood Estimation cannot be performed by standard optimization.

Luckily, tractable distributions on the Stiefel manifolds circumventing some of these problems have been brought up and studied over the last decades in the research field of directional statistics. The most well-studied of them is the von Mises–Fisher (vMF) distribution (also called the Matrix Langevin distribution in some papers) first introduced in [32], which is the one we will be using in this paper. Given a matrix-valued parameter $F\in R^{n\times p}$, the von Mises–Fisher distribution on the Stiefel Manifold is defined by its density: $p_{\mathrm{vMF}}\left(X\right)\propto \exp\left(\mathrm{Tr}\left(F^{⊤}X\right)\right)$. Written differently, if we denote by $f_{1},\ldots ,f_{p}$ the columns of *F* and by $x_{1},\ldots ,x_{p}$ those of *X*, we have

$$p_{\mathrm{vMF}}\left(X\right)\propto \exp\left(\langle f_{1},x_{1}\rangle +\ldots +\langle f_{p},x_{p}\rangle \right)\;.$$

In this expression, each $x_{i}$ is drawn toward $f_{i}/\left|f_{i}\right|$ (up to the orthogonality constraint). The norm $|f_{i}|$ can be interpreted as a concentration parameter that determines the strength of the attraction toward $f_{i}/\left|f_{i}\right|$. The von Mises–Fisher distribution can be considered analogous to a Euclidean Gaussian distribution with a diagonal covariance matrix: the density imposes no interaction between the components of *X*, so that the only dependency between the columns is the orthogonality constraint. The equivalent of the Gaussian mode (which is the same as the Gaussian mean) is given by the following lemma:

**Lemma** **1.** *The von Mises–Fisher distribution with parameter F reaches its maximum density value at $X=\pi_{V}\left(F\right)$, where $\pi_{V}$ is an orthogonal projection onto the Stiefel manifold.*

**Proof.**  From the definition of the von Mises–Fisher density, we have:

$$\begin{array}{cc} {\mathrm{argmax}}_{X^{⊤}X=I_{p}}\mathrm{Tr}\left(F^{⊤}X\right) & ={\mathrm{argmax}}_{X^{⊤}X=I_{p}}-\frac{1}{2}\mathrm{Tr}\left(F^{⊤}F\right)+\mathrm{Tr}\left(F^{⊤}X\right)-\frac{1}{2}\mathrm{Tr}\left(X^{⊤}X\right)\\ \; & ={\mathrm{argmin}}_{X^{⊤}X=I_{p}}\frac{1}{2}{∥F-X∥}^{2}\;, \end{array}$$

with $∥\cdot ∥$ the Frobenius norm. Hence, by definition, $\pi_{V}\left(F\right)$ maximizes the von Mises–Fisher density. Note that the projection onto the Stiefel manifold is not uniquely defined, as $V_{n,p}$ is not convex.  □

The following lemma allows us to compute such a projection.

**Lemma** **2.** *Let $M\in R^{n\times p}$, and $M=UDV^{⊤}$ ($U\in R^{n\times p},D\in R^{p\times p},V\in R^{p\times p}$) the Singular Value Decomposition of M. If M has full rank, then $UV^{⊤}$ is the unique projection of M onto the Stiefel manifold $V_{n,p}$.*

**Proof.**  Let us consider the Lagrangian related to the constrained optimization problem $\pi_{V}\left(M\right)\in {\mathrm{argmin}}_{X^{⊤}X=I_{p}}\frac{1}{2}{∥M-X∥}^{2}$:

$$L\left(X,\Lambda \right)=\frac{1}{2}{∥M-X∥}^{2}-\mathrm{Tr}\left(\Lambda^{⊤}\left(I_{p}-X^{⊤}X\right)\right)\;.$$

Then the Karush–Kuhn–Tucker theorem [33] shows that, if $X^{*}$ is a local extremum of $X\mapsto \frac{1}{2}{∥X-M∥}^{2}$ over $V_{n,p}$, then there exists $\Lambda^{*}$ such that $\nabla_{X}L\left(X^{*},\Lambda^{*}\right)=0$. This gradient writes:

$$\begin{array}{cc} \nabla_{X}L\left(X^{*},\Lambda^{*}\right) & =X^{*}-M+X^{*}\left(\Lambda^{*}+\Lambda^{*⊤}\right)\\ \; & =X^{*}\left(I+\Lambda^{*}+\Lambda^{*⊤}\right)-M=0\;. \end{array}$$

Since $X\in V_{n,p}$ and *M* has full rank, the symmetric matrix $\Omega =I+\Lambda^{*}+\Lambda^{*⊤}$ must be invertible, so that $X^{*}=M\Omega^{-1}$. Hence

$$I_{p}=X^{*⊤}X^{*}=\Omega^{-1}M^{⊤}M\Omega^{-1}\Leftrightarrow \Omega^{2}=M^{⊤}M=VD^{2}V^{⊤}\;.$$

The matrix square roots of $M^{⊤}M$ are exactly given by the Ω’s of the form $VRV^{⊤}$, with $R=\mathrm{Diag}(\pm D_{11},\ldots ,\pm D_{pp})$. We get $X^{*}=M\Omega^{-1}=UDR^{-1}V^{⊤}$, which gives the following objective function:

$$\begin{array}{cc} {∥M-X^{*}∥}^{2} & ={∥U\left(D-DR^{-1}\right)V^{⊤}∥}^{2}={∥D-DR^{-1}∥}^{2}\;. \end{array}$$

As *D* has a positive diagonal, this function is globally minimized by R=D, so that the unique projection is $X^{*}=UV^{⊤}$.  □

**Figure 1:**
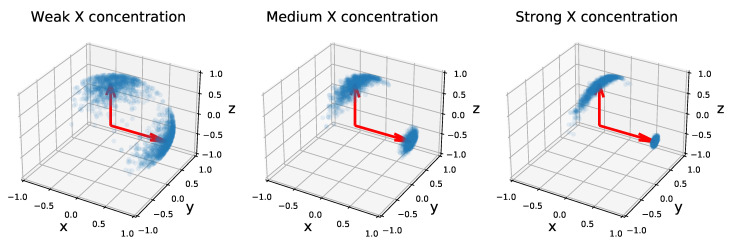
One thousand samples of three von Mises–Fisher distributions on $V_{3,2}$. The mode of the distribution is represented by two red arrows along the *x* and *z* axes, and the two vectors in each matrix by two blue points. The concentration parameters are set to $|f_{z}|=10$ and $|f_{x}|\;\in \;\left[10,100,500\right]$ (from **left** to **right**). Samples are drawn with an adaptive Metropolis–Hastings sampler using the transition kernel described in Section 4. A stronger concentration of the *x* vector impacts the spread of the *z* vector.

The simple, interpretable density of the von Mises–Fisher distribution comes with several important advantages. First, it allows using classical Markov Chain Monte Carlo (MCMC) methods to sample efficiently from the distribution (see Figure 1 for examples of distributions over $V_{3,2}$). Next, the form of the density makes it a member of the exponential family, which is a key requirement to perform latent variable inference with the MCMC-Stochastic Approximation Expectation-Maximization algorithm (MCMC-SAEM, [34]) used in this paper. Finally, reasonably efficient algorithms exist to perform Maximum Likelihood Estimation (MLE) of the parameter *F*. This point will be further developed in Section 4.

#### 2.2.3. Application to Network Modeling

Statistical modeling on the Stiefel manifold has proven relevant to analyze networks. By considering the matrix of the *p* eigenvectors associated with the largest eigenvalues of an adjacency matrix as an element of $V_{n,p}$, Hoff and colleagues [35,36,37,38] showed that probabilistic modeling of the eigenvector matrix on the Stiefel manifold provides a robust representation while allowing to quantify the uncertainty of each edge and estimate the probability of missing links. In these papers, the eigenvectors follow a uniform prior distribution. In the present study, we propose to model the eigenvectors of several networks as samples of a common distribution on $V_{n,p}$ concentrated around a mode.

## 3. A Latent Variable Model for Graph Data Sets

### 3.1. Motivation

We model graphs in a data set by studying the eigendecomposition of their adjacency matrices. Given such a symmetric weighted adjacency matrix $A\in R^{n\times n}$, the spectral theorem grants the existence of a unique decomposition $A=X\Lambda X^{⊤}=\sum_{i=1}^{r}\lambda_{i}x_{i}x_{i}^{⊤}$, where *r* is the rank of *A*, and $\lambda_{1}\geq \ldots \geq \lambda_{r}$ and $x_{1},\ldots ,x_{r}$ are the eigenvalues and the orthonormal eigenvectors of the matrix. This decomposition is unique up to the sign of the eigenvectors, as long as the non-zero eigenvalues values have multiplicity-one, which always holds in practice. The interest of this decomposition for graph adjacency matrices is threefold.

First, the eigendecomposition of the adjacency matrix reflects the modularity of a network, i.e., the extent to which its nodes can be divided into separate communities. For instance, in the case of the Stochastic Block Model (SBM), each node *i* is randomly assigned to one cluster c(i) among *p* possible ones. Nodes in clusters $c,c^{\prime }$ are connected independently with probability $P_{cc^{\prime }}$. In expectation, the adjacency matrix is equal to the matrix $\left(P_{c(i)c(j)}\right)$, which has the rank of *p* at most. In samples of the SBM as well as real modular networks, the decay of the eigenvalues allows estimating the number of clusters. The eigenvectors related to non-zero eigenvalues are used to perform clustering on the nodes to retrieve their labels.

Furthermore, this decomposition provides a natural expression of *A* as a sum of rank-one patterns $x_{i}x_{i}^{⊤}$. Modeling vectors as a weighted sum of patterns is at the core of dictionary learning-based and mixed effects models, which have proven of great interest to the statistics and machine learning research communities. In the specific case of graph data sets, such a model was recently proposed by D’Souza et al. [28] in the context of brain networks analysis. The authors learn a set of rank-one patterns without orthogonality constraints, and estimate the adjacency matrices as weighted sums of these patterns, in order to use the weights as regression variables. However, they consider the patterns as population-level variables only. This choice prevents taking into account potential individual-level variations.

Finally, the dominant eigenvectors yield strong patterns that are likely to remain stable among various networks in a data set, up to a certain variability. In other words, given *N* adjacency matrices $A^{\left(1\right)},\ldots ,A^{\left(N\right)}$ and their eigendecompositions $\left(X^{\left(1\right)},\Lambda^{\left(1\right)}\right)$, …, $\left(X^{\left(N\right)},\Lambda^{\left(N\right)}\right)$, the first columns of the $X^{\left(k\right)}$’s should remain stable among subjects (up to a column permutation and/or change of sign). On the contrary, smaller eigenvalues should be expected to correspond to eigenvectors with greater variability. The recent work of Chen et al. [39] takes stock of this remark to analyze the Laplacian matrices of brain networks (the Laplacian is a positive matrix that can be computed from the adjacency matrix). The authors propose to compute the $L^{1}$ mean of the $X^{\left(k\right)}$’s first *p* columns in order to get a robust average *X* representative of the population. As the $X^{\left(k\right)}$’s are composed of *p* orthonormal vectors, their average should have the same property: it ensures that the obtained matrix can be interpreted as a point that best represents the distribution. Its definition thus formulates as an optimization problem over the Stiefel manifold $V_{n,p}$. The authors show that taking this geometric consideration into account leads to better results than computing a Euclidean mean.

In the next section, we introduce our statistical analysis framework. We model the perturbations of the adjacency matrix eigendecomposition to account for the variability within a network data set.

### 3.2. Model Description

We propose to account for the variability in a set of networks by considering the random perturbation of both the patterns (*X* variable) that compose the networks and their weight (λ variable). In this study, we consider each pattern $x_{i}$ (column of *X*) and each weight $\lambda_{i}$ to be independent of one another. This assumption, although a first approximation, leads to a tractable inference problem and interpretable results. Future works could consider interactions between the $x_{i}$’s or the $\lambda_{i}$’s, as well as the dependency between both.

The model decomposition of each adjacency matrix $A^{\left(k\right)}$ in a data set writes

(1) $$A^{\left(k\right)}=X^{\left(k\right)}\mathrm{Diag}\left(\lambda^{\left(k\right)}\right)X^{(k)⊤}+\varepsilon^{\left(k\right)}$$

with $X^{\left(k\right)}$ a pattern matrix, $\lambda^{\left(k\right)}$ the pattern weight vector and $\varepsilon^{\left(k\right)}$ the symmetric residual noise. The $X^{\left(k\right)}$ and $\lambda^{\left(k\right)}$ are independent unobserved variables that determine the individual-level specificity of network *k*. We model these variables as follows:

(2) $$\left\{\begin{array}{c} X^{\left(k\right)}\overset{i.i.d}{∼}\mathrm{vMF}\left(F\right)\\ \lambda^{\left(k\right)}\overset{i.i.d}{∼}N\left(\mu ,\sigma_{\lambda }^{2}I_{p}\right)\\ \varepsilon^{\left(k\right)}\overset{i.i.d}{∼}N\left(0,\sigma_{\varepsilon }^{2}I_{n(n+1)/2}\right). \end{array}\right.$$

**Figure 2:**
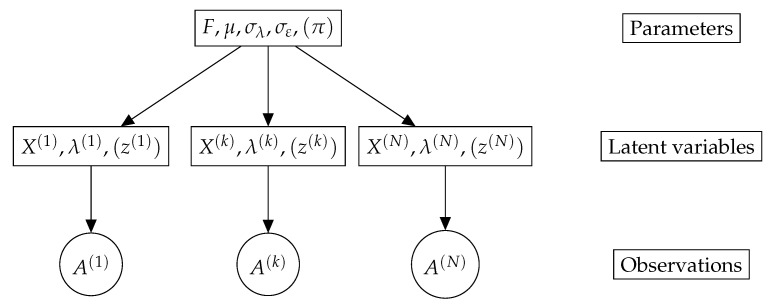
Graphical model for a data set of adjacency matrices $A_{1},\ldots ,A_{N}$. The variables π and $z^{\left(k\right)}$ can be added to get a mixture model.

The matrix $F\in R^{n\times p}$ parametrizes a von Mises–Fisher distribution for the eigenvectors matrix $X^{\left(k\right)}$, and the eigenvalues $\lambda^{\left(k\right)}$ follow a Gaussian distribution with mean $\mu \in R^{p}$ and independent components with variance $\sigma_{\lambda }^{2}$. We further impose that the columns of *F* are orthogonal: this constraint ensures that the maximum of the log-density $\langle f_{1},x_{1}\rangle +\ldots +\langle f_{p},x_{p}\rangle$ is reached at $\pi_{V}\left(F\right)=(f_{1}/|f_{1}|,\ldots ,f_{p}/|f_{p}\left|\right)$. In this model, the matrix $\pi_{V}\left(F\right)$ is the mode of the distribution of patterns and plays a role similar to the mean of a Gaussian distribution. The mode of the full distribution of latent variables thus refers to $\left(\pi_{V}\left(F\right),\mu \right)$. In the particular case where *F* has orthogonal columns, the column norms of *F* correspond to its singular values. In the remainder of the paper we call them the *concentration parameters* of the distribution. The variability of the adjacency matrices is thus fully characterized by $\sigma_{\varepsilon }$, $\sigma_{\lambda }$ and the concentration parameters. The pattern weights $\lambda^{\left(k\right)}$ are the eigenvalues of the $X^{\left(k\right)}\mathrm{Diag}\left(\lambda^{\left(k\right)}\right)X^{(k)⊤}$ term, and we thus call them eigenvalues even though they are not the actual spectrum of the real adjacency matrices $A^{\left(k\right)}$. Our model is summarized in Figure 2.

Note that this model may be adapted to deal with other types of adjacency matrices. The distribution for $\lambda^{\left(k\right)}$ can be effortlessly changed to a log-normal distribution to model data sets of positive matrices like covariance matrices. Binary networks can be modeled by removing the $\varepsilon^{\left(k\right)}$ noise and adding a Bernoulli sampling step, considering $X^{\left(k\right)}\lambda^{\left(k\right)}X^{(k)⊤}$ as a logit. Adjacency matrices with positive coefficients are considered by adding the softplus function $x\mapsto \log(1+e^{x})$ in Equation (1). These extensions bring a wide range of possible statistical models for adjacency matrices for which the estimation procedure is the same as the one developed below.

Equation (1) theoretically requires each $A^{\left(k\right)}$ to be close to a rank *p* matrix. While this assumption is reasonable for well-clustered networks like samples of an SBM, some real-life networks exhibit heavy eigenvalue tails and cannot be approximated accurately using low rank matrices. While our model should not be expected to provide a perfect fit on general networks data sets, its main goal is to retrieve the principal modes of variability and their weight in an interpretable way, comparable to probabilistic Principal Component Analysis (PCA) or probabilistic Independent Component Analysis (ICA) [40]. An important difference with these methods is that our model expresses each of the *p* components using only an *n*-dimensional vector, whereas PCA and ICA require an n×n matrix per component to model adjacency matrices.

In the case of well clustered networks, our model can be seen as a refinement of the SBM better suited to data sets of networks. The SBM is designed to handle one single network and mainly addresses the problem of identifying the communities. In the case of network data sets, all subjects share the same node labels and the communities can be more easily identified by averaging the edge weights over the subjects. The main assumption of the SBM that the connections between the nodes are independent of one another prevents from further analyzing individual-level variability. In contrast, our model can account for the impact of a node variation on its connections, as well as pattern variations affecting the whole network. In the limit where the concentration parameters become very large and the weight variance is small, the patterns become constant and our model becomes equivalent to an SBM for networks organized in distinct clusters.

Another remark can be made on the identifiability of the model: the manifold of matrices of the form $X\mathrm{Diag}\left(\lambda \right)X^{⊤}$ with $X\in V_{n,p},\lambda \in R^{p}$ (also known as the non-compact Stiefel manifold) has a tangent space *T* with dimension $\dim\left(V_{n,p}\right)+p=np-p\left(p-1\right)/2$ at $X^{\left(k\right)}\mathrm{Diag}\left(\lambda^{\left(k\right)}\right)X^{(k)⊤}$. The noise $\varepsilon^{\left(k\right)}$ can be decomposed into components in *T* and its orthogonal complement $T^{⊤}$ with dimension $n^{2}-np+p\left(p-1\right)/2$. The component in *T* thus induces an implicit source of variability on *X* and λ, which depends on $\sigma_{\varepsilon }$. We show in the experiment section that it may lead to underestimating the concentration parameters $\left(|f_{1}|,\ldots ,|f_{p}|\right)$. While aware of this phenomenon, we consider it an acceptable trade-off regarding the simple formulation of Equation (2).

### 3.3. Mixture Model

The matrix distribution introduced in the previous section can be integrated in a mixture model to account for heterogeneous populations with a multi-modal distribution and variability. It amounts to considering *K* clusters with, for each cluster, a probability $\pi^{c}$ and a parameter $\theta^{c}=\left(F^{c},\mu^{c},\sigma_{\varepsilon }^{c},\sigma_{\lambda }^{c}\right)$. The mixture model writes hierarchically:

(3) $$\left\{\begin{array}{c} z^{\left(k\right)}∼\mathrm{Categorical}\left(\pi \right)\\ \left(X^{\left(k\right)}|z^{\left(k\right)}=c\right)∼\mathrm{vMF}\left(F^{c}\right)\\ \left(\lambda^{\left(k\right)}|z^{\left(k\right)}=c\right)∼N\left(\mu^{c},{\left(\sigma_{\lambda }^{c}\right)}^{2}I_{p}\right)\\ \left(A^{\left(k\right)}|X^{\left(k\right)},\lambda^{\left(k\right)},z^{\left(k\right)}=c\right)∼N\left(X^{\left(k\right)}\mathrm{Diag}\left(\lambda^{\left(k\right)}\right)X^{(k)⊤},{\left(\sigma_{\varepsilon }^{c}\right)}^{2}I_{n(n+1)/2}\right). \end{array}\right.$$

We show in the next section on parameter estimation that the mixture layer only comes at a small algorithmic cost.

## 4. A Maximum Likelihood Estimation Algorithm

We now turn to the problem of estimating the model parameters $\theta =(F,\mu ,\sigma_{\lambda },\sigma_{\varepsilon })$ given a set of observations ${\left(A^{\left(k\right)}\right)}_{k=1}^{N}$. Let us denote $\lambda \cdot X=X\mathrm{Diag}\left(\lambda \right)X^{⊤}$. The complete likelihood is expressed as:

$$\begin{array}{cc} p(\left(A^{\left(k\right)}\right),\left(X^{\left(k\right)}\right),\left(\lambda^{\left(k\right)}\right);\theta ) & =\prod_{k=1}^{N}\frac{1}{K(\theta )}p\left(A^{\left(k\right)}|X^{\left(k\right)},\lambda^{\left(k\right)};\theta \right)p\left(X^{\left(k\right)};\theta \right)p\left(\lambda^{\left(k\right)};\theta \right) \end{array}$$

with

$$\left\{\begin{array}{c} p\left(A^{\left(k\right)}|X^{\left(k\right)},\lambda^{\left(k\right)};\theta \right)=\frac{1}{|\sigma_{\varepsilon }{|}^{n^{2}}{\left(2\pi \right)}^{n^{2}/2}}\exp\left[-\frac{1}{2\sigma_{\varepsilon }^{2}}{∥A^{\left(k\right)}-\lambda^{\left(k\right)}\cdot X^{\left(k\right)}∥}^{2}\right]\\ p\left(X^{\left(k\right)};\theta \right)=\frac{1}{C_{n,p}(F)}\exp\left[\mathrm{Tr}(F^{⊤}X^{\left(k\right)})\right]\\ p\left(\lambda^{\left(k\right)};\theta \right)=\frac{1}{|\sigma_{\lambda }{|}^{p}{\left(2\pi \right)}^{p/2}}\exp\left[-\frac{1}{2\sigma_{\lambda }^{2}}{∥\lambda^{\left(k\right)}-\mu ∥}^{2}\right] \end{array}\right.$$

We compute the maximum of the observed likelihood $p(\left(A^{\left(k\right)}\right);\theta )$ using the MCMC-SAEM algorithm introduced in the next section. The MLE is not unique, as a permutation or a change of sign in the columns of *X* (together with a permutation of λ) yield the same model. This invariance can be broken by sorting the eigenvalues μ in increasing order as long as they are sufficiently spread. However, in practice, several eigenvalues may be close, and imposing such an order hinders the convergence of the algorithm. We thus choose to leave the optimization problem unchanged and deal with the permutation invariance by adding a supplementary step to the MCMC-SAEM algorithm.

### 4.1. Maximum Likelihood Estimation for Exponential Models with the MCMC-SAEM Algorithm

When dealing with latent variable models, the standard tool for MLE is the Expectation-Maximization (EM) algorithm [41]. Given a general parametric model p(y,z;θ) with *y* an observed variable and *z* a latent variable, performing MLE amounts to maximizing logp(y;θ)=log∫p(y,z;θ)dz, which is intractable in practice with classical optimization routines. The EM algorithm allows indirectly maximizing this objective by looping over two alternating steps:

1. *E-step*: Using the current value of the parameter $\theta_{t}$, compute the expectation
  $$Q_{t}\left(\theta \right)=E_{p(z|y;\theta_{t})}\left[\log p\left(y,z;\theta \right)\right]\;;$$
2. *M-step*: Find $\theta_{t+1}\in {\mathrm{argmax}}_{\theta }Q_{t}\left(\theta \right)$.

While the EM algorithm proves efficient to deal with simple models like mixtures of Gaussian distributions, it requires adaptation for the cases of more complicated models where the expectation in the $Q_{t}\left(\theta \right)$ function is intractable, and the distribution $p(z|y,\theta_{n})$ cannot be explicitly sampled from to approximate the expectation.

The Markov Chain Monte Carlo–Stochastic Approximation EM algorithm (MCMC-SAEM) developed by [34] aims at overcoming these hurdles in the case of models belonging to the Curved Exponential Family. For such models, the log-density expresses as logp(y,z;θ)=〈S(y,z),φ(θ)〉+ψ(θ), where S(y,y) is a sufficient statistic. The $Q_{t}$ function then simply rewrites $Q_{t}\left(\theta \right)=\langle E_{p(z|y;\theta_{t})}\left[S\left(y,z\right)\right],\varphi \left(\theta \right)\rangle +\psi \left(\theta \right)$. In the MCMC-SAEM algorithm, the expectation of sufficient statistics is computed throughout iterations using Stochastic Approximation. The samples from $p(z|y;\theta_{t})$ are drawn using a MCMC kernel $q(z|z_{t};\theta_{t})$ with invariant distribution $p(z|y;\theta_{t})$. The procedure is recalled in Algorithm 1. Under additional assumptions on the model and the Markov kernel, the MCMC-SAEM algorithm converges toward a critical point of the initial objective logp(y;θ) [42,43].

In the case of the model proposed in this paper, the MCMC-SAEM is well suited to the problem at hand as we have to deal with a latent variable model. In a setting with manifold-valued latent variables, the E-step of the SAEM algorithm becomes intractable; using the MCMC-SAEM allows overcoming this hurdle. Following the outline of Algorithm 1, we need to draw samples from $p(X^{\left(k\right)},\lambda^{\left(k\right)}|A^{\left(k\right)};\theta )$ and perform the maximization step using the stochastic approximation of sufficient statistics.
**Algorithm 1:** The MCMC-SAEM Algorithm
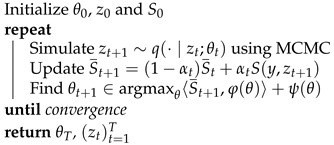


### 4.2. E-Step with Markov Chain Monte Carlo

#### 4.2.1. Transition Kernel

The target density $p(X^{\left(k\right)},\lambda^{\left(k\right)}|A^{\left(k\right)};\theta )$ is known up to a normalizing constant, and it is sufficient to use MCMCs based on the Metropolis–Hastings acceptance rule [44]. The MCMC is structured as a Gibbs sampler alternating simulations of $X^{\left(k\right)}$ and $\lambda^{\left(k\right)}$ for each individual. Note that conditional density $p(\lambda^{\left(k\right)}|X^{\left(k\right)},A^{\left(k\right)};\theta )$ is a Gaussian distribution. However, when experimenting with the MCMC-SAEM, we find that using Metropolis–Hastings-based transitions rather than sampling directly from the true conditional distribution accelerates the Markov chain convergence. This is why we perform a Metropolis–Hastings within Gibbs sampler for both variables [45]. We generate proposals for λ with a symmetric Gaussian kernel with adaptive variance in order to reach a target acceptance rate. We also use a Metropolis Hastings transition for *X*, with the constraint that the variable stays on the Stiefel manifold. Several techniques can be used to generate such proposals. The most natural equivalent of the symmetric random walk consists of a geodesic random walk generated by normally distributed tangent vectors. This method can be employed as the exponential map on the Stiefel manifold has a closed-form expression relying on the matrix exponential [30]. Another option is to use the curves given by the Cayley transform as in [46]: Cayley curves can be considered a fast first-order approximation of the exponential map. Finally, a more direct approach consists of making non-manifold Gaussian transitions and projecting the result back onto the manifold using Lemma 2. In our experiments these three approaches turn out to give very similar performances, and in practice we use the last method, which is also the fastest.

**Remark** **1.** *Our numerical implementation offers the possibility to use the Metropolis Adjusted Langevin Algorithm (MALA) instead of Metropolis–Hastings, as the gradient of the log-likelihood can be computed explicitly. While the experiments we have presented rely on the Metropolis–Hastings kernel, which is faster overall, we find that in some cases where the dimensions n and p grow large the MALA kernel allows accelerating the convergence.*

#### 4.2.2. Permutation Invariance Problem

The non-uniqueness of the MLE translates into a practical hurdle to the convergence of the MCMC: if two eigenvalues $\mu_{i},\mu_{j}$ are close, we get $\left(\mu_{i},\mu_{j}\right)\cdot \left(x_{i},x_{j}\right)≃\left(\mu_{j},\mu_{i}\right)\cdot \left(x_{i},x_{j}\right)$. As a consequence, the distribution $p(X^{\left(k\right)},\lambda^{\left(k\right)}|A^{\left(k\right)};\theta )$ is multi-modal in $X^{\left(k\right)}$, with a dominant mode close to $\pi_{V}\left(F\right)$ and other modes corresponding to column sign variations and permutations among similar eigenvalues. These modes are numerical artifacts rather than likely locations for the true value of $X^{\left(k\right)}$. Exploring them in the MCMC-SAEM hinders the global convergence: they encourage the samples to spread over the Stiefel manifold, which in turn yields a very bad estimation of *F* by inducing a bias toward the uniform distribution.

We address the permutation invariance problem by adding a column matching step every five SAEM iterations for the first third of the SAEM iterations. This step is a greedy algorithm that aims at finding the column permutation of a sample $X^{\left(k\right)}$ that makes it closest to $M=\pi_{V}\left(F\right)$. It proceeds recursively by choosing the columns $m_{i}$, $x_{j}$ with the greatest absolute correlation. The steps are summarized in Algorithm 2. The greedy permutation algorithm causes the MCMC samples to stabilize around a single mode, allowing estimation of the *F* parameter.
**Algorithm 2:** Greedy column matching
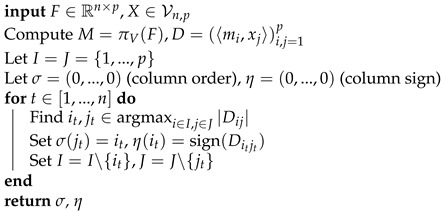


### 4.3. M-Step with Saddle-Point Approximations

The maximization step of the MCMC-SAEM algorithm has a closed form expression, except for the parameter *F*. In this section, we recall a method to estimate *F* in a general setting and apply this method to get the optimal model parameters given sufficient statistics.

#### 4.3.1. Maximum Likelihood Estimation of Von Mises–Fisher Distributions

The main obstacle to retrieving the parameter *F* given samples $X_{1},\ldots ,X_{N}$ is the normalizing constant of the distribution: though analytically known, it is hard to compute in practice (see Pal et al. [47] for a computation procedure when n=2). Jupp and Mardia [48] proved that the MLE exists and is unique as long as p<n and N≥2, or p=n and N≥3. Khatri and Mardia [32], who first studied the properties of the MLE, showed the following result:

**Theorem** **1** ([32])**.** *Let $X_{1},...,X_{N}$ be N samples from a von Mises–Fisher distribution and $\;\bar{\;X\;}\;=\frac{1}{n}\sum_{i=1}^{n}X_{i}$. Let $\;\bar{\;X\;}\;=\;\bar{\;U\;}\;\;\bar{\;D\;}\;\;\bar{\;V\;}{\;}^{⊤}$ be the Singular Value Decomposition (SVD) of $\;\bar{\;X\;}\;$. Then the Maximum Likelihood Estimator can be written under the form $\hat{F}=\;\bar{\;U\;}\;\mathrm{Diag}\left(\hat{s}\right)\;\bar{\;V\;}{\;}^{⊤}$, with $\hat{s}\in R_{+}^{p}$.*

Maximizing the log-likelihood of samples $X_{1},\ldots ,X_{N}$ is thus equivalent to solving the optimization problem

(4) $${\mathrm{argmax}}_{s\in R^{p}}\mathrm{Tr}\left[\;\bar{\;V\;}\;\mathrm{Diag}\left(s\right)\;\bar{\;U\;}{\;}^{⊤}\;\bar{\;X\;}\;\right]-\log C_{n,p}\left(\;\bar{\;U\;}\;\mathrm{Diag}\left(s\right)\;\bar{\;V\;}{\;}^{⊤}\right),$$

where $C_{n,p}\left(F\right)$ is the normalizing constant of the vMF distribution.

Several methods were proposed to solve this problem: the authors of [32] provide approximate formulas when the singular values of *F* are all either very large or very small. The authors of [49] propose a method to approximate the normalizing constant, which in turn yields a surrogate objective for the MLE giving satisfactory results. Finally, in [50], a different formula is proposed, which applies when the singular values are small. When experimenting with von Mises–Fisher distributions, we found that the method proposed by [49] gives the most robust results for a wide range of singular values of *F*, even in a high-dimensional setting.

#### 4.3.2. Application to the Proposed Model

Computational details for the likelihood rearrangement are deferred to Appendix A. The model belongs to the curved exponential family, and its sufficient statistics are:

$$S\left(A,X,\lambda \right)=\left\{\begin{array}{c} S^{1}=\frac{1}{N}\sum_{k=1}^{N}X^{\left(k\right)}\\ S^{2}=\frac{1}{N}\sum_{k=1}^{N}\lambda^{\left(k\right)}\\ S^{3}=\frac{1}{N}\sum_{k=1}^{N}{∥\lambda^{\left(k\right)}∥}^{2}\\ S^{4}=\frac{1}{N}\sum_{k=1}^{N}{∥A^{\left(k\right)}-\lambda^{\left(k\right)}\cdot X^{\left(k\right)}∥}^{2}\;. \end{array}\right.$$

These sufficient statistics are updated using the MCMC samples ${\left(X_{t}^{\left(k\right)},\lambda_{t}^{\left(k\right)}\right)}_{k=1}^{N}$ with the stochastic approximation $\;\bar{\;S\;}{\;}_{t+1}=\left(1-\alpha_{t}\right)\;\bar{\;S\;}{\;}_{t}+\alpha_{t}S\left(A,X_{t},\lambda_{t}\right)$. The optimization problem defined by the M-step of the SAEM algorithm gives the following results:

(5) $${\hat{\theta }}_{t}=\left\{\begin{array}{cc} \hat{F} & =\hat{F}\left(\;\bar{\;S\;}{\;}_{t}^{1}\right)\\ \hat{\mu } & =\;\bar{\;S\;}{\;}_{t}^{2}\\ {\hat{\sigma }}_{\lambda }^{2} & =\frac{1}{p}\left({∥\hat{\mu }∥}^{2}-2\langle \hat{\mu },\;\bar{\;S\;}{\;}_{t}^{2}\rangle +\;\bar{\;S\;}{\;}_{t}^{3}\right)\\ {\hat{\sigma }}_{\varepsilon }^{2} & =\frac{1}{n^{2}}\;\bar{\;S\;}{\;}_{t}^{4}\;, \end{array}\right.$$

where $\hat{F}\left(\;\bar{\;S\;}{\;}_{t}^{1}\right)$ denotes the MLE of the von Mises–Fisher distribution. As explained in the section above, the method proposed by Kume et al. [49] allows estimating the normalizing constant of general Fisher–Bingham distributions. The approximation relies on rewriting the constant to make it depend on a density that fits into the framework of Saddle-Point Approximations [51]. We recall the main steps of the computation procedure for this approximation in Appendix A for the specific, simple case of vMF distributions.

In the definition of our model, we impose that the columns of *F* are orthogonal. As recalled in Section 2.2, the MLE for the vMF mode is $\;\bar{\;M\;}\;=\;\bar{\;U\;}\;\;\bar{\;V\;}{\;}^{⊤}$, where $\;\bar{\;X\;}\;=\;\bar{\;U\;}\;\;\bar{\;D\;}\;\;\bar{\;V\;}{\;}^{⊤}$ is the SVD of the empirical arithmetic mean of samples. Since the column norms correspond to the singular values when the columns are orthogonal, the MLE under this constraint can be sought under the form $\;\bar{\;M\;}\;\mathrm{Diag}\left(s\right)$. Hence, the optimization problem is used to estimate *F*:

(6) $${\mathrm{argmax}}_{s\in R^{p}}\mathrm{Tr}\left[\mathrm{Diag}\left(s\right)\;\bar{\;M\;}{\;}^{⊤}\;\bar{\;X\;}\;\right]-\log{\hat{C}}_{n,p}\left(\;\bar{\;M\;}\;\mathrm{Diag}\left(s\right)\right)\;,$$

with ${\hat{C}}_{n,p}$ the approximation of the normalizing constant. We solve this optimization problem using the open source optimization library scipy.optimize.

The complete procedure is summarized in Algorithm 3.

### 4.4. Algorithm for the Mixture Model

The mixture model adds a cluster label $z^{\left(k\right)}$ for each subject and a list π of cluster probabilities. The model still remains in the curved exponential family, and the MCMC-SAEM algorithm can still be used. The Gibbs sampler now also updates $z^{\left(k\right)}$: it consists of sampling from the probabilities $p(z^{\left(k\right)}|X^{\left(k\right)},\lambda^{\left(k\right)},A^{\left(k\right)};\pi ,\theta )$, which can be computed explicitly. The sufficient statistics $S^{1},S^{2},S^{3},S^{4}$ are defined and stored for each cluster. The statistics of cluster *c* are updated using only the indices *k* such that $z^{\left(k\right)}=c$. The variable π adds new sufficient statistics: $S^{\pi }={\left(\#\left\{k|z^{\left(k\right)}=c\right\}/N\right)}_{c=1}^{K}$. The related MLE estimate of π is $\hat{\pi }=S^{\pi }$.

In our implementation, we initialize the clusters using the K-Means algorithm. We use the tempering proposed by [52] for the *z* sampling step in order to encourage points moving between clusters at the beginning of the algorithm. The vMF parameters $F^{c}$ are aligned every 5 SAEM iterations using Algorithm 2 in order to allow the latent variables to move between the regions of influence of different clusters through small Metropolis–Hastings steps. The resulting algorithm is detailed in Appendix C.

### 4.5. Numerical Implementation Details

We initialize the algorithm by taking the first eigenvectors and eigenvalues of each adjacency matrix. Algorithm 2 is used to align the eigenvectors between samples. In order to accelerate the convergence, we perform a small number of hybrid MCMC-SAEM steps at the start of the algorithm, where the MCMC step on *X* is replaced with a gradient ascent step on the log-likelihood. These first steps move the $X^{\left(k\right)}$’s to an area of $V_{n,p}$ with high posterior probability, which accelerate the convergence of the MCMC, as the *X* variable is the slowest to evolve along the MCMC-SAEM iterations. The Riemannian gradient ascent is detailed in Appendix B.
**Algorithm 3:** Maximum Likelihood Estimation algorithm for $\theta =(F,\mu ,\sigma_{\varepsilon },\sigma_{\lambda })$
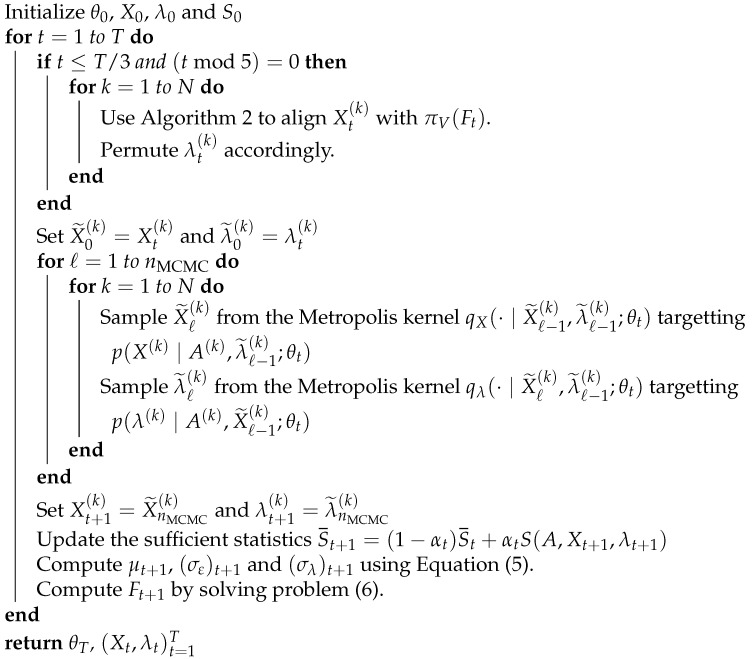


The Metropolis–Hastings transition variance is selected adaptively throughout the iterations using stochastic approximation. At SAEM step t+1, the proportion of accepted Metropolis transitions is computed. The logarithm of the variance is then incremented according to the rule $\log\sigma_{MH}^{t+1}=\log\sigma_{MH}^{t}+\ell_{t}/2t^{0.6}$, with $\ell_{t}=\pm 1$ depending on whether the proportion of accepted jumps should be increased or decreased.

During the first half of the *T* iterations we set $\alpha_{t}=1$ in order to minimize the impact of poor initializations. Then $\alpha_{t}$ decreases as $1/{\left(t-T/2\right)}^{0.6}$, which ensures the theoretical convergence of the algorithm.

The algorithms as well as all the experiments presented in this paper are implemented with Python 3.8.6. The package Numba [53] is used to accelerate the code. We provide a complete implementation (https://github.com/cmantoux/graph-spectral-variability, accessed on 19 April 2021), which allows reproducing the experiments on synthetic data and running the algorithm on new data sets.

## 5. Experiments

### 5.1. Experiments on Synthetic Data

#### 5.1.1. Parameters Estimation Performance

First we investigate the ability of the algorithm to retrieve the correct parameters when the data are simulated according to Equations (1) and (2). We test the case (n=3,p=2), referred to as low-dimensional, where *X* can be visualized in three dimensions as well as the case (n=40,p=20), referred to as high-dimensional.

##### Small Dimension

**Figure 3:**
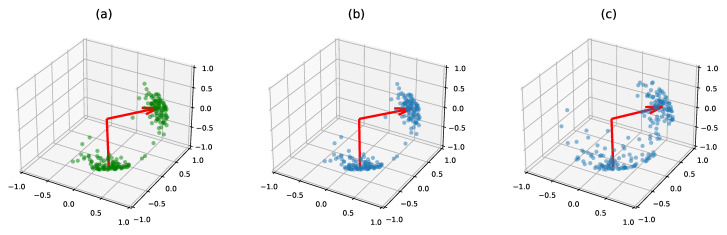
True latent variables $X^{\left(k\right)}$ and their posterior MCMC mean estimation. The red arrows represent the true $\pi_{V}\left(F\right)$ parameter and its estimate $\pi_{V}\left(\hat{F}\right)$. (**a**) The true mode and samples. (**b**) Mode and samples estimates when $\sigma_{\varepsilon }=0.1$. (**c**) Mode and samples estimates when $\sigma_{\varepsilon }=4$. The columns are rearranged using Algorithm 2 to ease visualization. The latent variables are accurately estimated when the noise is small. A stronger noise causes the estimated latent variables to spread over the Stiefel manifold.

We choose *F* with two orthogonal columns uniformly in $V_{3,2}$ with column norms (25,10). Using these low concentration parameters makes the results simple to visualize. We set μ=(20,10) and $\sigma_{\lambda }=2$, and generate N=100 matrices $A^{\left(k\right)}$ with $\sigma_{\varepsilon }=0.1$ and 100 other matrices with the same $X^{\left(k\right)}$’s and $\lambda^{\left(k\right)}$’s but a much stronger noise standard deviation $\sigma_{\varepsilon }=4$. We run the MCMC-SAEM algorithm for 100 iterations with 20 MCMC steps for each maximization step. The results are shown in Figure 3. In both cases, the mode of the vMF distribution $\pi_{V}\left(F\right)$ is well recovered. In the small noise case, the posterior *X* samples closely match the true *X* samples, and the estimated concentration parameters (23.7,8.0) remain close to ground truth. In the strong noise case, the posterior samples spread much farther around $\hat{F}$ than the true samples: the estimated concentration is (9.9,2.8). This result highlights the remark in Section 3.2 on the bias induced by the Gaussian noise on the latent variable spread: the best *X* variable to estimate the matrix $A^{\left(k\right)}$ is moved apart from the true $X^{\left(k\right)}$ in a random direction because of the noise $\varepsilon^{\left(k\right)}$ living outside the manifold.

##### High Dimension

**Figure 4:**
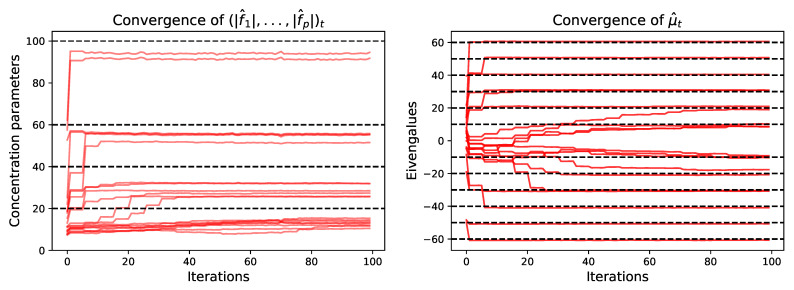
Convergence of the concentration parameters $\left(|f_{1}|,\cdots ,|f_{p}|\right)$ (**left**) and the mean eigenvalues μ (**right**) over the SAEM iterations. The red lines represent the values of the parameters along the iterations. The black dotted lines represent the true values, which are grouped in batches to ease visualization. The convergence is fastest for the large eigenvalues and concentration parameters. At the start of the algorithm, the biggest changes in the parameters come from the greedy permutation performed every 5 iterations. As explained in the text, the concentration parameters are underestimated. However, they keep the right order of magnitude, which allows interpreting the output of the algorithm in practice.

**Figure 5:**
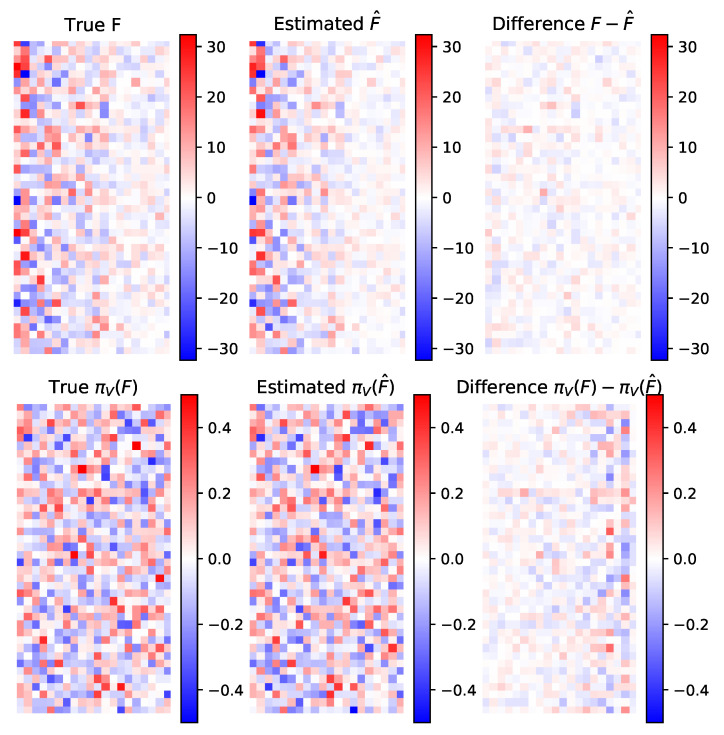
Von Mises-Stiefel distribution parameter *F* and its estimation $\hat{F}$. (**Top row**): the two parameters and their difference. (**Bottom row**): mode of the true distribution (given by $\pi_{V}\left(F\right)$), mode of the estimated distribution $\pi_{V}\left(\hat{F}\right)$ and their difference. The images show each matrix as an array of coefficients, with pixel color corresponding to coefficient amplitude. Since the matrix columns are orthonormal, the projection just consists of normalizing the columns. The columns are sorted by decreasing the concentration parameter. The normalized columns of *F* corresponding to the smallest concentration parameters are estimated with less precision.

We now consider a synthetic data set of N=200 samples generated from 20 latent patterns in dimension 40 and $\sigma_{\varepsilon }=1,\sigma_{\lambda }=2$, with various sizes of concentration parameters and eigenvalues, pairing large eigenvalues together with high concentrations. We run the MCMC-SAEM algorithm for 100 iterations with 20 MCMC steps per SAEM iteration to obtain convergence. The convergence of the parameters is shown in Figure 4. For both the concentration parameters and the eigenvalues, the algorithm starts by finding the highest values, only identifying lower values progressively afterward. The lowest values are associated to patterns with low weight, hence their recovery is naturally more difficult. As in the previous sections, the concentration parameters tend to be underestimated, indicating wider spreading around the mode vectors $f_{i}/\left|f_{i}\right|$ than the original latent variable. However, the ordering and orders of magnitude of the concentrations stay coherent, which, in practice, allows interpreting them and comparing them to each other. The estimation $\hat{F}$ matches the true parameter with a relative Root Mean Square Error (rRMSE) of 28%. As can be seen in Figure 5, the estimated normalized columns closely correspond to the original ones except when the concentration parameters get too small to allow for a good estimation, as explained above.

**Figure 6:**
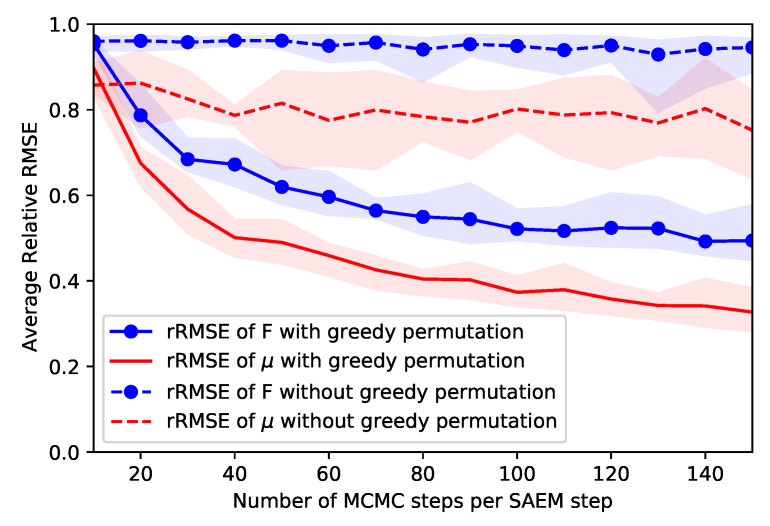
Relative RMSE of parameters *F* and μ after 100 MCMC-SAEM iterations depending on the number of MCMC steps per SAEM iteration. Results are averaged over 10 experiments to reduce the variance. The shaded areas indicate the extremal values across the repeated experiments. When using the greedy permutation, the rRMSE decreases rapidly when the number of MCMC steps increases before stabilizing. On the other hand, without the permutation step, the performance stays poor for any number of MCMC steps per maximization, as the parameters cannot be estimated correctly. In this experiment only, the latent variables are initialized at random to highlight the result.

We use this example to illustrate the role of the algorithm hyperparameters on the practical convergence, namely the number of MCMC steps per SAEM iteration and the column matching step. We consider the same data set, but we initialize the MCMC-SAEM algorithm with random latent variables instead of the method described in Section 4: this worst-case initialization highlights the differences between the settings more easily. It is also closer to the case of real data sets: the MCMC and model parameters are slower to converge on real data, as the adjacency matrices are not actual samples of the theoretical model distribution. For different numbers of MCMC steps per SAEM iterations, we run the MCMC-SAEM algorithm for 200 iterations 10 times to average out the random initialization dependency, with and without the column matching step. Then we compute the relative RMSE of the parameters *F* and μ at the end of the algorithm. The rRMSE averaged over the 10 runs is shown in Figure 6. It can be seen that when the column matching step is used, increasing the number of MCMC steps at a fixed number of SAEM iterations improves the estimation. It allows accelerating the convergence, as MCMC steps are faster than the maximization step (which requires repeated vMF normalizing constant computations). However, when the number of MCMC steps gets too large, the performance improvement stagnates while the execution time increases. We find that, in practice, using between 20 and 40 MCMC steps per SAEM iterations is a good compromise in terms of convergence speed. Figure 6 also illustrates the need for the column matching step proposed in Section 4: when not used, the parameters hardly converge to the right values, even with a large number of MCMC steps per SAEM iteration. When the eigenvectors are permuted differently across the samples, the related eigenvalues cannot be estimated accurately, as they mix together when averaged in the maximization step. The abscence of permutations also spreads the eigenvectors over the Stiefel manifold, which prevents estimating the von Mises–Fisher parameter. Since Algorithm 2 is very fast to execute, it is not a computational bottleneck. In our experiments, the number of SAEM iterations between successive column permutation steps did not have a significant impact as long as it was not too high: values between 5 and 20 produced similar results.

##### Model Selection

In all the experiments on simulated data presented in this paper, we use the correct number of columns *p*, which we assume to be known. However, when studying real data sets, classical model selection procedures like the Bayesian Information Criterion cannot be applied to our model: they require computing the complete probability of the observations $p\left(A|\theta_{m}\right)=\int_{V_{n,p}}\int_{R^{p_{m}}}p\left(A|X,\lambda ,\theta_{m}\right)\;dX\;d\lambda$ for each model $\theta_{m}$. This probability cannot be computed explicitly, as it requires integrating over the Stiefel manifold, which results in intractable expressions using the matrix hypergeometric function [49].

In practice, several tools can be used to choose the number of latent patterns. First, the marginal likelihood p(A∣X,λ;θ) or the error $∥A-\lambda \cdot X∥$ can be used to evaluate the model expressiveness. As *p* increases, the error will naturally diminish and should be very small for p=n. As with linear models, the proportion of the variance captured by λ·X can be computed to evaluate the improvement gained by adding new patterns. The concentration parameters of the von Mises–Fisher distribution also give important information on pattern relevance: if a pattern has a very low concentration parameter, it means that the related eigenvectors are widely spread across the Stiefel manifold. Smaller concentrations are thus related to overfitting, as they do not correspond to actual patterns contributing to the data set variability. The relative importance of concentration parameters can be compared numerically with the vMF concentration obtained on samples from the uniform distribution gathered with Algorithm 2.

**Remark** **2.** *In this paper, we approximate the posterior mean of MCMC samples of $X^{\left(k\right)}$ by projecting their arithmetic mean over the Stiefel manifold. We find this procedure a very convenient alternative to computing the Fréchet mean (i.e., the Riemannian center of mass) over the manifold for two reasons. First, computing the Fréchet mean requires an extensive use of the Riemannian logarithm. Although a recent paper [31] allows computing this logarithm, the proposed algorithm heavily relies on matrix logarithm computations and requires points to remain very close to the mean. Similar iterative algorithms to compute the mean based on other retraction and lifting maps than the Riemannian exponential and logarithm were proposed and analyzed in [54], but in our experiments, these alternatives also turn out to require samples close to the mean point, especially in high dimensions. Second, projecting the mean sample onto the Stiefel manifold amounts to computing the mode of a vMF distribution. As shown in Appendix D, the vMF distribution is symmetric around its mode, which makes this mode a summary variable similar to the Gaussian mean.*

#### 5.1.2. Missing Links Imputation

Once the parameters $\hat{\theta }$ are estimated from adjacency matrices $A_{1},\ldots ,A_{N}$, missing links can be inferred on a new adjacency matrix *A*. Suppose that only a subset Ω of the edge weights is known: the weights of masked edges $\;\bar{\;\Omega \;}\;$ can be obtained by considering the posterior distribution $p(A_{\;\bar{\;\Omega \;}\;}|A_{\Omega };\theta )$. This distribution is obtained as a marginal of the full posterior $p(A_{\;\bar{\;\Omega \;}\;},X,\lambda |A_{\Omega };\theta )$. Sampling from this distribution yields a posterior mean as well as confidence intervals for the value of missing links. In the case of binary networks, the posterior distribution gives the probability of a link existing for each masked edge. Samples are obtained by Gibbs sampling using the same method as in Section 4. We also compute the Maximum A Posteriori (MAP) by performing gradient ascent on the posterior density of $\left(A_{\;\bar{\;\Omega \;}\;},X,\lambda \right)$ given $A_{\Omega }$.

**Figure 7:**
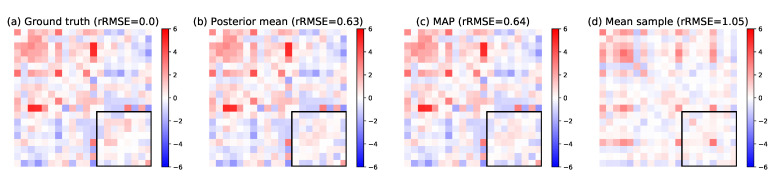
Result for missing link inference using the posterior distribution. (**a**) Ground truth input matrix *A*. (**b**) Posterior mean of the masked coefficients. (**c**) MAP estimator. (**d**) Mean of model samples for comparison. The area of masked edges is highlighted by a black square. Above each matrix is the rRMSE with the ground truth. Both the posterior mean and the MAP give a reasonable estimation for the missing weights, significantly better than the empirical mean of all adjacency matrices, which is the base reference for missing data imputation. The images show each matrix as an array of coefficients, with pixel color corresponding to coefficient amplitude.

We generate a synthetic data set of N=200 adjacency matrices with n=20 nodes and p=5. The noise level $\sigma_{\varepsilon }$ is chosen such that the average relative difference between the coefficients of $A^{\left(k\right)}$ and $\lambda^{\left(k\right)}\cdot X^{\left(k\right)}$ is 25%. We estimate the model parameters using the MCMC-SAEM algorithm. Then, we generate another 200 samples from the same model. We mask 16% of the edge weights corresponding to the interactions between the last eight nodes. The posterior estimation is compared with the ground truth for one matrix in Figure 7. Both the MAP and posterior mean allow to estimate the masked coefficients better than the mean sample $(A_{1}+\ldots +A_{N})/N$, which is the base reference for missing data imputation. They achieve, respectively, 58% (±28%) and 57% (±24%) rRMSE on average, whereas the mean sample has an 85% (±10% over the data set) relative difference to the samples on average. Finally, we perform the same experiment except we select the masked edges uniformly at random, masking 40% of the edges. This problem is easier than the former despite the larger amount of hidden coefficients because the missing connections are not aligned with each other. The posterior mean and the MAP achieve, respectively, 34% (±9% over the data set) and 35% (±7%) rRMSE, against 75% (±5%) for the mean sample.

Link prediction has been a very active research topic in network analysis for several decades, and numerous methods can be employed to address this problem depending on the setting [3,55,56]. However, the most commonly used approaches are designed to perform inference on a single network or consider the nodes as permutation invariant. In turn, the new approach we propose allows for population-informed prediction and uncertainty quantification. It could be used in practice to compare specific connection weights of a new subject with their distribution given the other coefficients and the population parameters. This comparison provides a tool to detect anomalies in the subject’s connectivity network stepping out of the standard variability.

**Remark** **3.** *The error uncertainties reported in this paper refer to the variance of the estimation error across the adjacency matrices.*

#### 5.1.3. Clustering on Synthetic Data

As explained in Section 3.3, our model can be used within a mixture to account for multi-modal distributions of networks. When experimenting with the clustering version of our algorithm on data sets with distinctly separated clusters, we noticed that the algorithm provides results similar to running K-Means and estimating the parameters on each K-Means cluster separately. However, the clusters in complex populations often overlap, and the ideal case where all groups are well separated rarely occurs. In this section, we show two examples of simulated data sets where the variabilities of the clusters makes them hard to distinguish with the sole application of the K-Means algorithm.

##### Small Dimension

**Figure 8:**
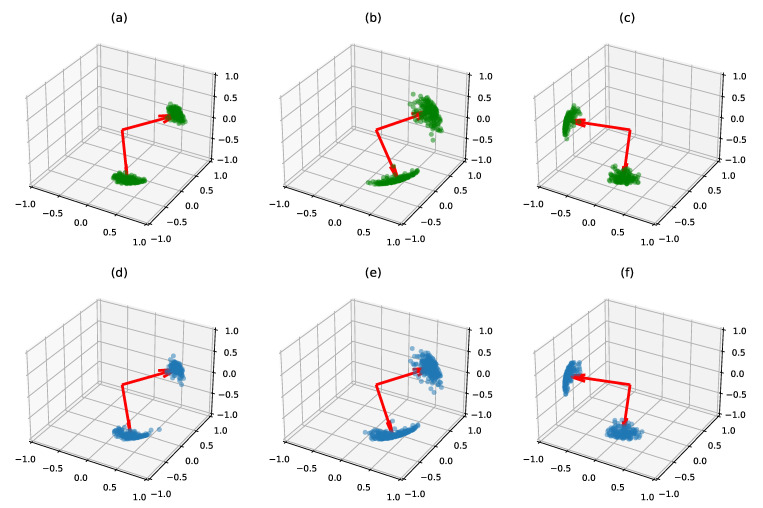
True latent variables $X^{\left(k\right)}$ and their posterior mean estimation for the clustering problem. (**Top row**): the plots (**a**–**c**) represent the true vMF modes (in red), as well as the true $X^{\left(k\right)}$ samples (in green) in their true class. (**Bottom row**): the plots (**d**–**f**) represent the three estimated vMF central modes (in red) and the estimated $X^{\left(k\right)}$ in their estimated class (in blue). The cluster centers are well recovered, as well as the concentration parameters. In particular, the two first clusters, which mainly differ by their concentration parameters, are correctly separated.

We test the mixture model estimation in the small dimensional case (n=3,p=2) where results can be visualized. We simulate three clusters of matrices as in Section 5.1.1 with N=500 samples overall. In order to make the problem difficult, we use the same mean eigenvalues for two clusters. We set the Stiefel modes of these clusters to be very close, differing mainly by their concentration parameters. We run the tempered MCMC-SAEM for 1000 iterations with a decreasing temperature profile $T_{t}=1+50/t^{0.6}$. Once the convergence is achieved, the estimated clusters are mapped to the true clusters. The eigenvalue parameters are estimated accurately with 2% rRMSE. The original and estimated von Mises–Fisher distributions are compared in Figure 8. We can see that each cluster distribution is well recovered. In particular, the overlapping distributions of cluster 1 and 2 are separated, and the higher concentration of cluster 1 is recovered in the estimation. This example also highlights the relevance of the MCMC-SAEM clustering procedure compared with its K-Means initialization: up to a label permutation, 50.4% of the K-Means proposed labels are correct, whereas the posterior distribution $p(z^{\left(k\right)}|A^{\left(k\right)};\hat{\theta })$ computed with the final MCMC samples predicts the correct answer for 79.6% of the model samples.

##### Larger Dimension

We now test the mixture model on a synthetic data set of 500 samples in dimension (n=20,p=10). We generate four clusters with Stiefel modes close to one another, with equal concentration parameters. The modes mainly differ by their mean eigenvalues $\mu^{c}$. The eigenvalue standard deviation $\sigma_{\lambda }$ is set to be of the same order of magnitude as the means μ, larger than most of its coefficients. The resulting data set is hard to estimate with classical clustering: the K-Means algorithm retrieves 53.6% of correct labels at best. In contrast, running the tempered MCMC-SAEM algorithm for 1000 iterations yields 99.4% of correct labels. The algorithm achieves this result by identifying the template patterns of each cluster despite the large variation in their weights. Once these template patterns are learned, the proportion of correctly classified samples increases and the mean eigenvalues of each cluster converge to a good estimation.

##### Model Selection

Selecting the number of clusters *K* is a known problem adressed for general mixture models [57]. Although it is well understood for simple Gaussian mixture models or for low dimensional data, other cases remain challenging problems. For the model proposed in this paper, likelihood-based procedures cannot be applied, as the complete likelihood is an integral over the Stiefel manifold (see Section 5.1.1). As with the selection of parameter *p*, the concentration parameters and the reconstruction errors could be used to choose the number of clusters. Using a *K* that is too small will result in stretching the latent von Mises–Fisher distributions toward low concentration parameters and large reconstruction errors. The reconstruction error should decrease slower once the right number of clusters has been reached.

**Remark** **4.** *The link prediction procedure described in Section 5.1.2 could also be applied in the mixture model to infer the coefficients of new networks of which the class is unknown.*

### 5.2. Experiments on Brain Connectivity Networks

**Figure 9:**
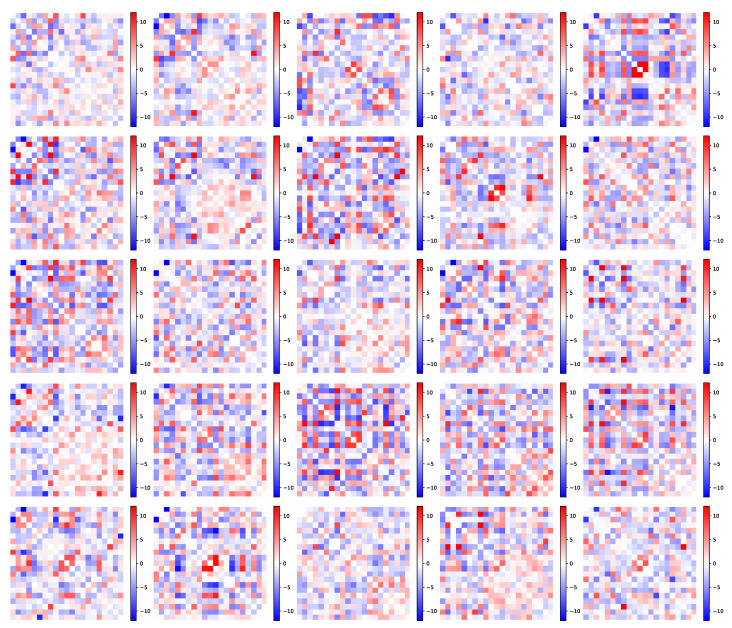
Functional connectivity matrices (21×21 ) of 25 UK Biobank subjects. The connectivity structure changes a lot depending on the subject, with various patterns expressing with different weights. The matrices in the data set have no diagonal coefficients; hence, the diagonals are shown as zero.

We test our model on the UK Biobank data repository [58]. The UK Biobank is a large scale data collection project, gathering brain imaging data on more than 37,000 subjects. In this paper, we are interested more specifically in the resting-state functional Magnetic Resonance Imaging data (rs-fMRI). The rs-fMRI measures the variations of blood oxygenation levels (BOLD signals) across the whole brain while the subject is in a resting state, i.e., receives no stimulation. The brain is then divided into regions through a spatial ICA that maximizes the signal coherence within each region [59]. Smaller regions give more detail on the brain structure but are less consistent across individuals. Finally, the raw imaging data are processed to obtain a matrix that gathers the temporal correlations between the mean blood oxygenation levels in each region. This matrix thus represents the way brain regions activate and deactivate with one another. It is called the *functional connectivity network* of the brain, as it provides information on the role of the regions rather than their physical connections. In the UK Biobank data used in the present study, the connectivity matrices are defined on a parcellation of the brain into n=21 regions. These connectivity matrices illustrate our purpose well: as shown in Figure 9, the data set has a very large diversity of networks that express in patterns with varying weights.

#### 5.2.1. Parameter Estimation

We run our algorithm on N=1000 subjects for 1000 SAEM iterations with 20 MCMC steps per SAEM iteration. Working on a restricted number of samples allows for a fast convergence toward the final values. Indeed, we noticed that, while most of the parameters stabilize relatively fast, the time to convergence of the concentration parameters grows with the number of samples. Apart from these concentration parameters, we obtained very similar results when taking all the UK Biobank subjects. In this section, we consider a decomposition into p=5 patterns. In Appendix E.1, we show the results obtained by taking different values of *p*.

**Figure 10:**
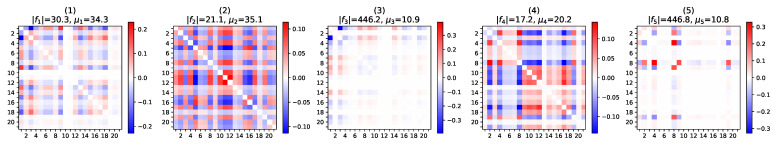
Normalized rank-one connectivity patterns. The matrix *i* represents $\mathrm{sign}\left(\mu_{i}\right)f_{i}f_{i}^{⊤}/{∥f_{i}∥}^{2}$. The caption above each pattern gives the related concentration parameter and mean eigenvalue. The diagonal coefficients are set to zero, as they do not correspond to values in the data set. The images show each matrix as an array of coefficients, with pixel color corresponding to coefficient amplitude.

In Figure 10, we show the *p* normalized patterns $f_{i}f_{i}^{⊤}/{∥f_{i}∥}^{2}$ obtained once the algorithm has converged. Patterns 3 and 5 have very high concentration parameters and only use a small subset of the nodes. The three other patterns have smaller concentration parameters. However, these concentrations are still high enough for the related columns of *X* to be significantly more concentrated than a uniform distribution: the average Euclidean distance between these three columns of $X^{\left(k\right)}$ and the related mode columns is 1.1 (±0.2 over the data set). Comparatively, the average distance between two points drawn uniformly on the Stiefel manifold is 2.4 (±0.2) (over 10,000 uniform samples).

**Figure 11:**
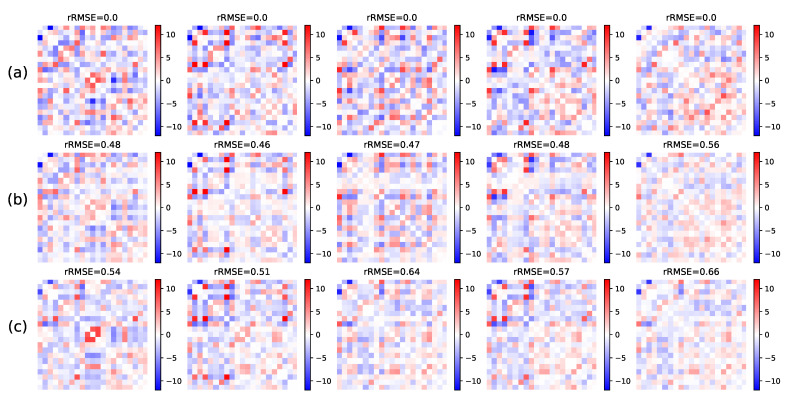
(**a**) UK Biobank connectivity matrices for 5 subjects. (**b**) Corresponding posterior mean value of λ·X estimated by the MCMC-SAEM. (**c**) Projection of the true connectivity matrices onto the subspace of the first five PCA components. The posterior mean matrix achieves a better rRMSE than PCA by capturing the main patterns of each individual matrix. As in Figure 10, the diagonal cofficients are set to zero.

Figure 11 displays data set matrices $A^{\left(k\right)}$ alongside the respective mean posterior estimates of $\lambda^{\left(k\right)}\cdot X^{\left(k\right)}$. For comparison purpose, we also compute the approximation given by the projection onto the subspace of the first five PCA components of the full data set, where each component has been vectorized. The λ·X matrices capture the main structure, whereas the PCA approximation relying on the same number of base components provides a less accurate reconstitution. Quantitatively, the λ·X term has a 47% (±5% over the data set) relative distance to *A*, whereas the PCA approximation has a 92% (±12%) relative distance to *A*. The λ·X representation accounts for 60% of the total variance, whereas the corresponding PCA representation only accounts for 35%. This difference highlights the benefits of taking into account the variations of the patterns across individuals. In a classical dictionary-based representation model, the patterns do not vary among individuals. In contrast, accounting for the pattern variability only adds a small number of parameters (one per pattern) and increases the representation power.

#### 5.2.2. Pattern Interpretation

Once the patterns are identified, they can be interpreted based on the function of the related involved brain regions. All brain regions can be found on a web page of the UK Biobank project (https://www.fmrib.ox.ac.uk/datasets/ukbiobank/group_means/rfMRI_ICA_d25_good_nodes.html, accessed on 19 April 2021). The regions analyzed in this section can be visualized on brain cuts in Appendix E.2.

Pattern 3 mainly represents the anti-correlation between regions 1 and 3. Region 1 comprises, among others, the inner part of the orbitofrontal cortex and the precuneus. These regions are parts of the Default Mode Network (DMN) of the brain, which is a large-scale functional brain network known to be active when the subject is at rest or mind-wandering [60]. Region 3 comprises part of the insular cortex and the post-central gyrus, which both play a role in primary sensory functions. The anti-correlation between regions 1 and 3 is a consequence of external sensations activating the sensory areas and decreasing the DMN activity. This anti-correlation is also one of the strongest coefficients in pattern 1.

Pattern 5 mainly features the dependency between nodes 2, 4, 8, 9, and 19, which are all related to the visual functions. Node 2 represents the parts of the occipital and temporal lobes forming the ventral and dorsal streams: they are theorized to process the raw sensory vision and hearing to answer the questions “what?” and “where?” [61]. Region 4 features the cuneus, which is a primary visual area in the occipital lobe. Region 8 spans over the whole occipital lobe, covering primary visual functions and associative functions like the recognition of color or movement. Region 9 comprises the continuation of the ventral and dorsal streams of region 2 in the parietal and medial temporal areas. Finally, Region 19 represents the V1 area that processes the primary visual information. Pattern 5 involving these regions has a very high concentration parameter, which means that this structure remains very stable among the subjects.

Considering that the subject’s activity in the MRI scanner mainly consists of looking around and laying still, it is coherent that the most stable patterns (i.e., with highest concentration parameters) during the resting-state fMRI measurement are the activity of the vision system and the anti-correlation between the DMN and sensory areas.

Pattern 4 also shows the interaction between the visual areas 2, 4, 8, and 19. It also includes the strong correlation between nodes 9, 10, 11, 12, and 17. Regions 10, 11, and 12 are involved in motor functions. Region 10 features part of the pre-central gyrus, which is central in the motor control function, and part of the post-central gyrus, which is involved in sensory information processing. Region 11 encompasses the entire pre-central gyrus. Region 12 includes a part of the motor and pre-motor cortex in the frontal lobe and the insular cortex. It also includes the cerebellum, which plays an important role in motor control, and the insular cortex, which also acts on the motor control, for instance, in the face and hands motion control [62]. Region 17 comprises the medial face of the superior temporal gyrus and the hippocampus, which are involved in short and long-term memory and spatial navigation.

Pattern 2 combines, to some extent, the structure contained in patterns 4 and 5. It features, among others, interactions between the visual areas and the correlation between the motor function areas.

**Remark** **5.** *The results and interpretation we present in this experiment depend on the state of the subjects—in this case, a resting state—and the brain parcellation used to obtain the definition of the regions. If we were to analyze another data set of subjects performing a different task, the connectivity patterns X would likely differ from their resting-state counterpart. It follows from the fact that two different phenomenons naturally require two different base dictionaries. Analyzing the pattern difference would thus provides a way to interpret the structure difference between the two settings. For instance, the role of the occipital lobe in the vision-involved patterns would likely change for tasks related to vision. However, if the brain regions are defined differently in the two experiments, the comparison can only be made in a qualitative way.*

#### 5.2.3. Link Prediction

**Figure 12:**
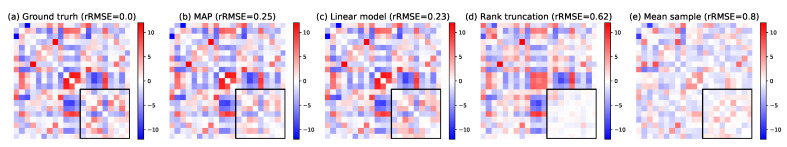
From left to right: (**a**) True connectivity matrix *A*. (**b**) MAP estimator for the masked coefficients framed in a black square. (**c**) Linear model prediction for the masked coefficients. (**d**) Rank 5 truncation of the matrix *A* with masked coefficients set to zero. (**e**) Mean of all data set matrices. Above each matrix is the rRMSE with the ground truth.

We evaluate the relevance of our model on fMRI data by testing the missing link imputation method introduced in the previous section. First we fit the model on N=1000 subjects. Then we take 1000 other test subjects and mask the edges corresponding to the interactions between the last nine nodes (except the diagonal coefficients, which are unknown and thus considered null). We compute the MAP estimator of the masked coefficients. For comparison purposes, we perform a linear regression to predict the masked coefficients given the visible ones. Finally, we truncate the matrix with masked coefficients to only keep the *p* dominant eigenvalues. This technique is at the core of low-rank matrix completion methods [63], and it relates naturally with the estimation derived from our model relying on low-rank variability. The result is shown for one sample in Figure 12. The linear model and the MAP estimator give comparable estimates, both close to the true masked coefficients. Over the 1000 test subjects, these estimators achieve on average 58% (±14% over the samples) rRMSE for the linear model and 65% (±15%) rRMSE for the MAP. Interestingly, our model uses only np+p+2=112 degrees of freedom, whereas the linear prediction model has dimension 26,640 and was specifically trained for the regression purpose.

Our model captures a faithful representation of the fMRI data set and uses far fewer coefficients than other models like PCA and linear regression by accounting for the structure of the interactions between the network nodes. It provides an explanation of the network variability using simple interpretable patterns, which correspond to known specific functions and structures of the brain. The variations of these patterns and their weight allow for a representation rich enough to explain a significant proportion of the variance and impute the value of missing coefficients.

## 6. Conclusions

This paper introduces a new model for the analysis of undirected graph data sets. The adjacency matrices are expressed as a weighted sum of rank-one matrix patterns. The individual-level deviations from the population average translate into variations of the patterns and their weight. Sample graphs are characterized by these variations in a way similar to PCA. The form of the decomposition allows for a simple interpretation: each pattern corresponds to a matrix with rank one and is thus represented by a vector of node coefficients. The variability of this decomposition is captured within a small number of variance and concentration parameters.

We use the MCMC-SAEM algorithm to estimate the model parameters as well as the individual-level variable. The parameter of von Mises–Fisher distributions is recovered by estimating the vMF normalizing constant, which allows retrieving both the mode and its concentration parameters. Future work could further investigate the role of the approximation error induced by the use of saddle-point approximations, comparing its performance with a recently proposed alternative method [64]. The impact of noise on the underestimation of the vMF distribution concentration also requires further analysis.

Experiments on synthetic data show that the algorithm yields good approximations of the true parameters and covers the posterior distributions of the latent variables. Our model can be used to infer the value of masked or unknown edge weights once the parameters are estimated. In practice, the posterior distribution could be compared to the real connections to detect anomalous connections that step out of the expected individual variability.

The model we introduce is a hierarchical generative statistical model, which easily extends to mixture models. We show that a mixture of decomposition models can be estimated with a similar algorithmic procedure and allow disentangling between modes of variability that are indistinguishable by a traditional clustering method.

We demonstrate the relevance of the proposed approach for the modeling of functional brain networks. Using few parameters, it explains the main components of the variability. The induced posterior representation is more accurate than PCA and gives a link prediction performance similar to a linear model, which has a comparably simple structure, but requires far more coefficients and was trained specifically to that purpose. The estimated connectivity patterns have a simple structure and lead to an interpretable representation of the functional networks. We show that our model identifies specific patterns for the visual information processing system or the motor control. The related concentration parameters allow measuring the variability of the function of the related brain regions across the subjects.

This work focuses on cross-sectional network data sets, i.e., populations where each adjacency matrix belongs to a different subject and is independent of the others. Our model could also be used as a base framework for longitudinal network modeling using the tools proposed by Schiratti et al. [65]. This would consist of considering time-dependent latent variables *X* and λ for each subject, evolving close to a population-level reference trajectory in the latent space.

Future work could investigate the dependencies between the latent variables of the model. Correlation can be introduced between the patterns by using Fisher–Bingham distributions on the Stiefel manifold [38] and between pattern weights with full Gaussian covariance matrices. Another direction to develop is the quantification of the uncertainty: by adding prior distributions on *F* and μ, a Bayesian analysis would naturally provide posterior confidence regions for the model parameters [47]. Finally, our framework could be adapted to model graph Laplacian matrices instead of adjacency matrices. The analysis of the eigenvalues and eigenvectors of the graph Laplacian has proven of great theoretical [66] and practical [67] interest in network analysis. Understanding the variability of the eigendecomposition of graph Laplacians could help to design robust models relying on spectral graph theory.

## Figures and Tables

**Figure A1:**
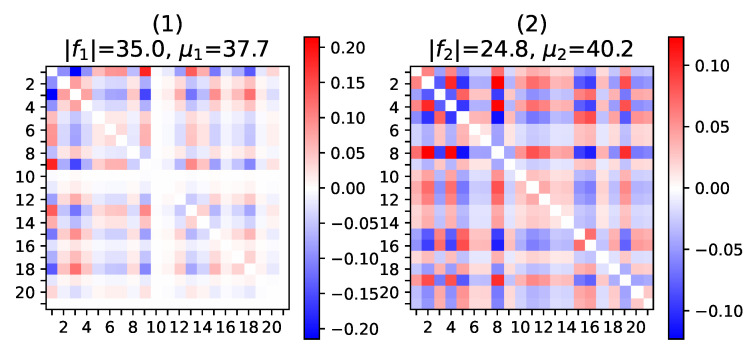
Normalized connectivity patterns when p=2, computed as in Figure 10.

**Figure A2:**
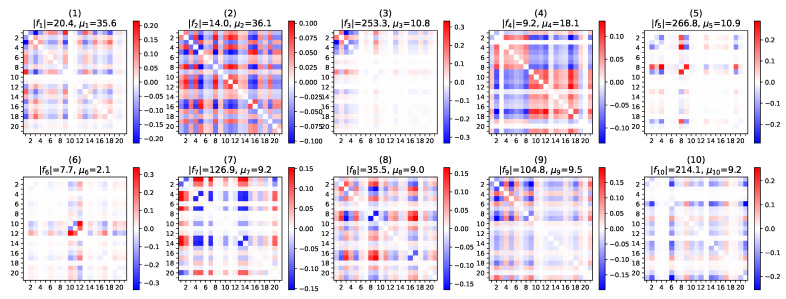
Normalized connectivity patterns when p=10, computed as in Figure 10.

**Figure A3:**
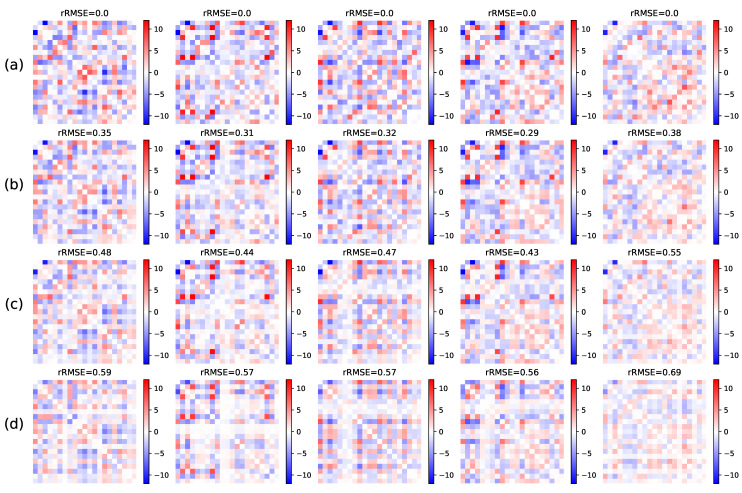
(**a**) UK Biobank connectivity matrices for 5 subjects. (**b**) M10 posterior mean value of λ·X. (**c**) M5 posterior mean value of λ·X. (**d**) M2 posterior mean value of λ·X. The rRMSE coherently increases as *p* decreases.

**Figure A4:**
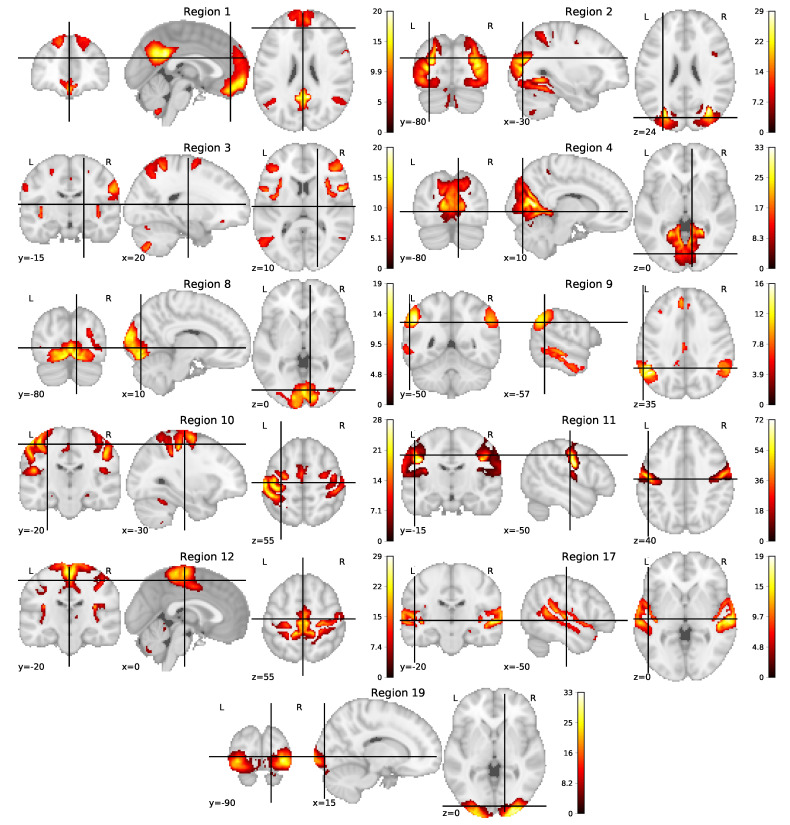
Frontal, sagittal, and transverse cuts of the brain for the UK Biobank fMRI brain regions analyzed in this paper. As explained in Section 5.2, region 1 comprises part of the Default Mode Network of the brain, which characterizes its activity at rest. Region 3, which is anti-correlated to region 1, is related to sensory functions. Regions 2, 4, 8, 9, and 19 are involved in the visual functions. Regions 10, 11, 12 correspond to motor control. Region 17 is involved in memory and spatial navigation. The L/R letters distinguish the left and right hemispheres. The black axes on each view give the three-dimensional position of the cut. The color strength corresponds to the truncated ICA weight.

## Data Availability

The data used in this paper come from the UK Biobank repository. The website is hosted at https://www.ukbiobank.ac.uk/ (accessed on 19 April 2021). The data are accessed upon application and cannot be made available publicly.

## Appendix A. SAEM Maximization Step

### Appendix A.1. Maximum Likelihood Estimates for μ, $\sigma_{\lambda }^{2}$, $\sigma_{\varepsilon }^{2}$

Up to a constant normalization term *c*, the complete log-likelihood of the model in the Gaussian case writes:

$$\begin{array}{cc} \log p(\left(A^{\left(k\right)}\right),\left(X^{\left(k\right)}\right),\left(\lambda^{\left(k\right)}\right);\theta ) & =\sum_{k=1}^{N}\log p\left(A^{\left(k\right)},X^{\left(k\right)},\lambda^{\left(k\right)};\theta \right)\\ \; & =\sum_{k=1}^{N}\left[-\frac{1}{2\sigma_{\varepsilon }^{2}}{∥A^{\left(k\right)}-\lambda^{\left(k\right)}\cdot X^{\left(k\right)}∥}^{2}-\frac{1}{2\sigma_{\lambda }^{2}}{∥\lambda^{\left(k\right)}-\mu ∥}^{2}+\mathrm{Tr}\left(F^{⊤}X^{\left(k\right)}\right)\right]\\ \; & \;\;-Nn^{2}\log\sigma -Np\log\sigma_{\lambda }-N\log C_{n,p}\left(F\right)+c\\ \; & =N\left[\mathrm{Tr}\left(F^{⊤}S_{1}\right)+\langle S_{2},\frac{1}{2\sigma_{\lambda }^{2}}\mu \rangle -S_{3}\frac{1}{2\sigma_{\lambda }^{2}}-S_{4}\frac{1}{2\sigma_{\varepsilon }^{2}}+\Psi \left(\theta \right)\right]\; \end{array}$$

with $\Psi \left(\theta \right)=-n^{2}\log\sigma_{\varepsilon }-p\log\sigma_{\lambda }-\log C_{n,p}\left(F\right)+c$, and 

$$\left\{\begin{array}{c} S^{1}=\frac{1}{N}\sum_{k=1}^{N}X^{\left(k\right)}\\ S^{2}=\frac{1}{N}\sum_{k=1}^{N}\lambda^{\left(k\right)}\\ S^{3}=\frac{1}{N}\sum_{k=1}^{N}{∥\lambda^{\left(k\right)}∥}^{2}\\ S^{4}=\frac{1}{N}\sum_{k=1}^{N}{∥A^{\left(k\right)}-\lambda^{\left(k\right)}\cdot X^{\left(k\right)}∥}^{2} \end{array}\right.$$

The model thus belongs to the curved exponential family, and its sufficient statistics are given by $\left(S^{1},S^{2},S^{3},S^{4}\right)$. The log-likelihood is componentwise convex in μ, $\sigma_{\lambda }^{2}$ and $\sigma_{\varepsilon }^{2}$. Computing its gradient yields one single critical point, which is thus the maximum value.

In the case of the binary model, the log-likelihood writes:

$$\begin{array}{cc} \log p(\left(A^{\left(k\right)}\right),\left(X^{\left(k\right)}\right),\left(\lambda^{\left(k\right)}\right);\theta ) & =\sum_{k=1}^{N}\sum_{i,j=1}^{n}\left[A_{ij}^{\left(k\right)}\log h{\left(\lambda^{\left(k\right)}\cdot X^{\left(k\right)}\right)}_{ij}+\left(1-A_{ij}^{\left(k\right)}\right)\log\left(1-h\right){\left(\lambda^{\left(k\right)}\cdot X^{\left(k\right)}\right)}_{ij}\right]\\ \; & \;+\sum_{k=1}^{N}-\frac{1}{2\sigma_{\lambda }^{2}}{∥\lambda^{\left(k\right)}-\mu ∥}^{2}+\mathrm{Tr}\left(F^{⊤}X^{\left(k\right)}\right)\\ \; & \;-Np\log\sigma_{\lambda }-N\log C_{n,p}\left(F\right)+c \end{array}$$

with h(x)=1/(1+exp(−x)) the sigmoid function, which applies component-wise on matrices. The distribution (A∣λ,X) is non parametric and needs no estimation. Hence, for all the model parameters $F,\mu ,\sigma_{\lambda }$ the MLE remains unchanged.

### Appendix A.2. Saddle-Point Approximation of $C_{n,p}\left(F\right)$

We recall the main steps to compute the approximation of $C_{n,p}\left(F\right)$ proposed by Kume et al. [49]. For more details on the justification of the approximation and applications to more general distributions, we refer the reader to the original paper. Our implementation provides a function spa.log_vmf, which computes this approximation. The approximation ${\hat{C}}_{n,p}\left(F\right)$ for von Mises–Fisher distributions is written in Equation (16) of [49]:

(A2) $${\hat{C}}_{n,p}\left(F\right)=\frac{2^{p}{\left(2\pi \right)}^{np/2-p(p+1)/4}}{|{\hat{K}}^{\prime \prime }{|}^{1/2}{\left|\hat{C}\right|}^{1/2}}\exp\left\{\frac{1}{2}\mathrm{vec}{\left(F\right)}^{⊤}{\hat{C}}^{-1}\mathrm{vec}\left(F\right)-\sum_{i=1}^{p}{\hat{\vartheta }}_{ii}\right\}\exp\left(T-p/2\right)\;.$$

Using the following definitions:

- $C\left(\vartheta \right)=-2I_{np}-2\sum_{1\leq i\leq j\leq p}\vartheta_{ij}\left(J_{ij}+J_{ji}\right)$. The matrix $J_{ij}$ is composed of $p^{2}$ blocks. Block (i,j) is the identity $I_{n}$ and all the other blocks are set to zero.
- $K\left(\vartheta \right)=-\frac{1}{2}\log\left|C\left(\vartheta \right)\right|-\frac{1}{2}\mu^{⊤}C{\left(\vartheta \right)}^{-1}\mu -\frac{1}{2}\mathrm{vec}{\left(\mu \right)}^{⊤}\mathrm{vec}\left(\mu \right)$. In this formula, μ is the n×p diagonal matrix with diagonal *p* singular values ω of *F*. The function K(ϑ) is the cumulant generating function.
- $\hat{\vartheta }$ is the unique solution of the so-called saddle-point equation $K^{\prime }\left(\vartheta \right)=\vartheta$. It has the explicit expression $\hat{\vartheta }=-1/\left(2\mathrm{Diag}\left(\hat{\phi }\right)\right)$, with ${\hat{\phi }}_{r}=\left(n+\sqrt{n^{2}+4\omega_{r}^{2}}\right)/\left(2\omega_{r}^{2}\right)$
- $\hat{C}$ is given by $C(\hat{\vartheta })$ and ${\hat{K}}^{\prime \prime }$ by $K^{\prime \prime }\left(\hat{\vartheta }\right)$.
- ${\hat{K}}^{\prime \prime }$ can be computed explicitly:
  $${\hat{K}}_{\left(r_{1},s_{1}\right),\left(r_{2},s_{2}\right)}^{\prime \prime }=\left\{\begin{array}{cc} 0 & r_{1}\neq r_{2}\;\mathrm{or}\;s_{1}\neq s_{2},\\ n{\hat{\phi }}_{r}{\hat{\phi }}_{s}+{\hat{\phi }}_{r}{\hat{\phi }}_{s}\left(\omega_{r}^{2}{\hat{\phi }}_{r}+\omega_{s}^{2}{\hat{\phi }}_{s}\right) & r_{1}=r_{2}<s_{1}=s_{2},\\ 2n{\hat{\phi }}_{r}^{2}+4\omega_{r}^{2}{\hat{\phi }}_{r}^{3} & r_{1}=r_{2}=s_{1}=s_{2} \end{array}\right.$$
- The parameter *T* is defined in Equation (8) of [49] and computed in the supplementary material of the paper in the case of vMF distributions. In first approximation, *T* can be considered zero.

As in the original paper, we validate our implementation by comparing the result with the Monte Carlo estimate of the normalizing constant produced by uniform sampling on the Stiefel manifold.

**Remark** **A1.**
*The −p/2 factor comes from using $B=-I_{n\times p}/2$ (and thus $V=I_{n\times p}$) and compensating with Equation (22) of [49] to handle vMF distributions, which otherwise have B=0. This point is not stated explicitly in the main text of the paper but it is explained in the related MATLAB implementation provided by the authors.*

## Appendix B. Gradient Formulas

The MCMC-SAEM initialization heuristic, as well as the MALA transition kernel, require the gradients of the log-likelihood with respect to the latent variables. In this section, we compute these gradients for the model with Gaussian perturbation and the model with binary coefficients.

### Appendix B.1. Model with Gaussian Perturbation

Consider the log-likelihood for the variables of only one subject (X,λ,A). Using the formula in Equation (A1), we can compute its gradients with respect to *X* and λ. For λ, it writes:

$$\begin{array}{c} \nabla_{\lambda }\log p\left(X,\lambda ,A;\theta \right)=-\left(\frac{1}{\sigma_{\varepsilon }^{2}}+\frac{1}{\sigma_{\lambda }^{2}}\right)\lambda +\frac{1}{\sigma^{2}}{\left(x_{i}^{⊤}Ax_{i}\right)}_{i=1}^{p}+\frac{1}{\sigma_{\lambda }^{2}}\mu \;, \end{array}$$

with ${\left(x_{i}\right)}_{i=1}^{p}$ the columns of *X*. Similarly, the Euclidean gradient for *X* is given by

$$\begin{array}{c} \nabla_{X}\log p\left(X,\lambda ,A;\theta \right)=\frac{1}{\sigma_{\varepsilon }^{2}}AX\mathrm{Diag}\left(\lambda \right)+F+4X\mathrm{Diag}\left(\lambda \right)X^{⊤}X\mathrm{Diag}\left(\lambda \right) \end{array}$$

Following Edelman et al. [30], the Riemannian gradient on the Stiefel manifold is then given by:

$$\begin{array}{c} \nabla_{X}^{V}\log p\left(X,\lambda ,A;\theta \right)=\nabla_{X}\log p\left(X,\lambda ,A;\theta \right)X^{(k)⊤}-X\nabla_{X}\log p\left(X,\lambda ,A;\theta \right) \end{array}$$

### Appendix B.2. Binary Model

Similarly, the log-likelihood gradients can be derived for the binary model. Let ${\tilde{x}}_{i}$ be the *i*-th *row* of *X* and ⊙ denote the entrywise product. We have:

$$\begin{array}{cc} \nabla_{\lambda }\log p\left(X,\lambda ,A;\theta \right) & =-\frac{1}{\sigma_{\lambda }^{2}}\left(\lambda -\mu \right)+\sum_{i,j=1}^{n}\left[A_{ij}h\left(-{\left(\lambda \cdot X\right)}_{ij}\right)-\left(1-A_{ij}\right)h\left({\left(\lambda \cdot X\right)}_{ij}\right)\right]\left({\tilde{x}}_{i}⊙{\tilde{x}}_{j}\right)\\ \nabla_{X}\log p\left(X,\lambda ,A;\theta \right) & =F+\sum_{i\neq j}\left[A_{ij}h\left(-{\left(\lambda \cdot X\right)}_{ij}\right)-\left(1-A_{ij}\right)h\left({\left(\lambda \cdot X\right)}_{ij}\right)\right]H_{ij}\\ \; & \;\;+\sum_{i=1}^{n}\left[A_{ii}h\left(-{\left(\lambda \cdot X\right)}_{ii}\right)-\left(1-A_{ii}\right)h\left({\left(\lambda \cdot X\right)}_{ii}\right)\right]K_{i}\;. \end{array}$$

In the latter formula, $H_{ij}$ is a n×p matrix with zeros everywhere except the *i*-th row equal to $\lambda ⊙{\tilde{x}}_{j}$ and the *j*-th row equal to $\lambda ⊙{\tilde{x}}_{i}$. $K_{i}$ is the n×p matrix with zeros everywhere except the *i*-th row equal to $2\lambda ⊙{\tilde{x}}_{i}$.

## Appendix C. Algorithm for the Clustering Model

We summarize in Algorithm A1 the procedure to estimate the MLE of a mixture model.

**Algorithm A1:** Maximum Likelihood Estimation of $\theta =(F,\mu ,\sigma_{\varepsilon },\sigma_{\lambda },\pi )$ for the mixture model

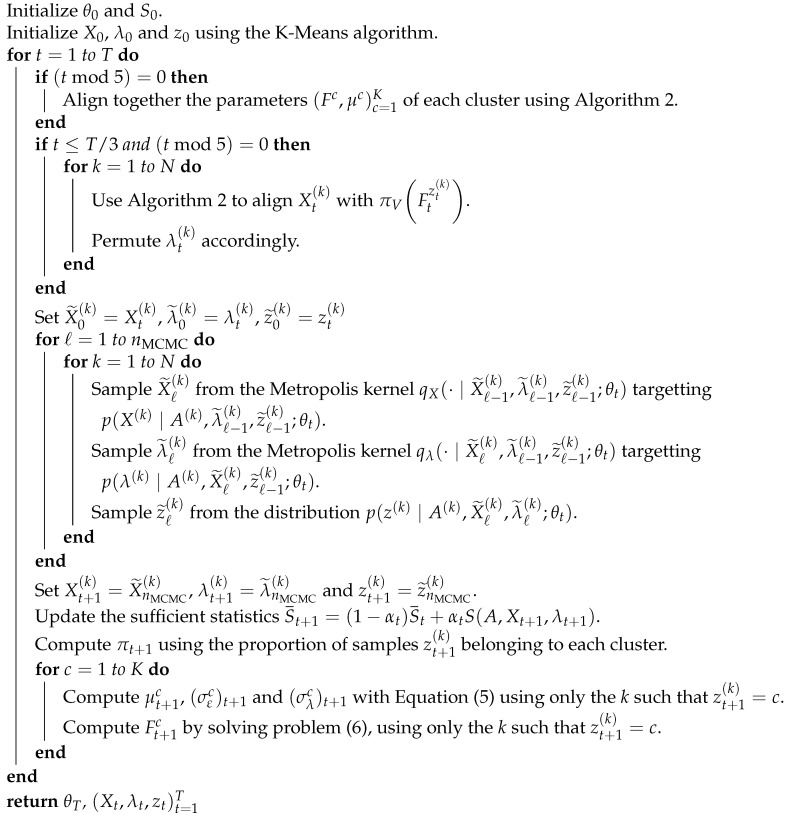

## Appendix D. Symmetry of Von Mises–Fisher Distributions

Let *F* be the parameter of a von Mises–Fisher distribution. Let $\exp_{X}$ be the Riemannian exponential map at *X*. We have the following result:

**Proposition** **A1.**
*Suppose that the columns of F are orthogonal. Let $M=\pi_{V}\left(F\right)$ be the mode of the vMF distribution and $D\in T_{M}V_{n,p}$ a tangent vector at M. Then $p_{\mathrm{vMF}}\left(\exp_{M}\left(D\right)\right)=p_{\mathrm{vMF}}\left(\exp_{M}\left(-D\right)\right)$, i.e., the vMF distribution is symmetric around its mode.*

**Proof.** Since the columns of *F* are orthogonal, we can write F=MΛ with $M=\pi_{V}\left(F\right)\in V_{n,p}$ and Λ=Diag(λ). Let $D\in T_{M}V_{n,p}$. As proven in [30], the geodesic $X_{t}$ starting at *M* with $X^{\prime }\left(0\right)=D$ is then given by

$$X_{t}=\left(M,M_{⊥}\right)\exp\left(tK_{M}\left(D\right)\right)I_{n,p}\;,$$

where exp is the matrix exponential, $M_{⊥}\in V_{n,n-p}$ is such that $M^{⊤}M_{⊥}=0$ and $K_{M}\left(D\right)$ is skew-symmetric: $K_{M}{\left(D\right)}^{⊤}=-K_{M}\left(D\right)$. Therefore, the von Mises–Fisher log-density along $X_{t}$ writes as:

$$\begin{array}{cc} \mathrm{Tr}(F^{⊤}X_{t}) & =\mathrm{Tr}(\Lambda M^{⊤}\left(M,M_{⊥}\right)\exp\left(tK_{M}\left(D\right)\right)I_{n,p})\\ \; & =\mathrm{Tr}(\Lambda I_{p,n}\exp\left(tK_{M}\left(D\right)\right)I_{n,p})\\ \; & =\mathrm{Tr}(I_{n,p}^{⊤}\exp{\left(tK_{M}\left(D\right)\right)}^{⊤}I_{p,n}^{⊤}\Lambda^{⊤})\\ \; & =\mathrm{Tr}(I_{p,n}\exp\left(tK_{M}{\left(D\right)}^{⊤}\right)I_{n,p}\Lambda )\\ \; & =\mathrm{Tr}(\Lambda I_{p,n}\exp\left(-tK_{M}\left(D\right)\right)I_{n,p})\\ \; & =\mathrm{Tr}(F^{⊤}X_{-t}) \end{array}$$

Therefore the von Mises–Fisher density is symmetric around its mode. □

## Appendix E. Additional Details on the UK Biobank Experiment

### Appendix E.1. Impact of the Number p of Patterns

We perform the same experiment as in Section 5.2 with different numbers of patterns, p∈{2,5,10}, always running the MCMC-SAEM for 1000 iterations with 20 MCMC steps per SAEM iteration. We call the related models M2, M5, and M10. The normalized patterns of M2 and M10 are reproduced in Figure A1 and Figure A2. The patterns of M2 correspond to patterns 1 and 2 of M5 and M10. Similarly, the patterns of M5 correspond to patterns 1 to 5 of M10. This result confirms that our model acts in a way comparable to PCA, selecting first the dominant patterns with the largest eigenvalues. Figure A3 compares the posterior means of λ·X given by M2, M5 and M10 for 5 subjects. Coherently, the approximation $\lambda^{\left(k\right)}\cdot X^{\left(k\right)}$ refines and gets closer to $A^{\left(k\right)}$ as *p* increases. Over the 1000 subjects, these posterior means achieve, respectively, 57% (±7%), 47% (±5%) and 35% (±4%) relative RMSE.

However, this observation does not assess whether higher values of *p* provide additional relevant features to represent the network structure. The following result illustrates this idea. We repeat the experiment of missing link MAP imputation on models M2 and M10. We find that both M2 and M10 yield a worse prediction than M5 on this task: model M2 gets 70% (±16%) rRMSE and M10 gets 76% (±16%) rRMSE, whereas model M5 gets 65% (±15%) rRMSE. While the prediction performance of M2 is expected to be worse than M5’s, observing a worse prediction performance in M10 means that the information captured by the additional components does not help infer the network structure. As with PCA, the components with lesser amplitude are less relevant to perform regression tasks; this idea is at the core of Partial Least Square Regression [68].

Therefore, the parameter *p* should be chosen with care when using our model for predictive purposes. The experiment presented above can be used to quantify the relevance of the obtained representation, but other methods could be explored. Future work could investigate the question of parameter selection by adapting Bayesian model selection methods to our method, as well as likelihood ratio tests or criteria like BIC and AIC.

### Appendix E.2. Brain Regions of the UK Biobank fMRI Correlation Networks

As explained in Section 5.2, the Regions Of Interest (ROIs) that define the correlation networks are detected automatically using a spatial ICA [59]. Each component of the ICA attributes a weight to each brain voxel. The brain regions are visualized by selecting the voxels with weight above a certain threshold. The obtained level set may be scattered over the brain, which sometimes makes their interpretation difficult. In Figure A4, we show the brain regions mentioned in the interpretation of the patterns identified by our model, namely regions 1, 2, 3, 4, 8, 9, 10, 11, 12, 17, 19. In this figure, as well as online, the ICA weight threshold value is set to 5.
